# Transcriptome analysis of Sézary syndrome and lymphocytic-variant hypereosinophilic syndrome T cells reveals common and divergent genes

**DOI:** 10.18632/oncotarget.27120

**Published:** 2019-08-20

**Authors:** Andrea M. Moerman-Herzog, Daniel A. Acheampong, Amanda G. Brooks, Suzan M. Blair, Ping-Ching Hsu, Henry K. Wong

**Affiliations:** ^1^ Department of Dermatology, University of Arkansas for Medical Sciences, Little Rock, Arkansas, USA; ^2^ Joint Graduate Program in Bioinformatics, University of Arkansas at Little Rock and University of Arkansas for Medical Sciences, Little Rock, Arkansas, USA; ^3^ Fay W. Boozman College of Public Health, University of Arkansas for Medical Sciences, Little Rock, Arkansas, USA

**Keywords:** Sézary syndrome, lymphocytic-variant hypereosinophilic syndrome, biomarkers, microarrays, progression

## Abstract

Sézary syndrome (SS) is an aggressive cutaneous T cell lymphoma with pruritic skin inflammation and immune dysfunction, driven by neoplastic, clonal memory T cells in both peripheral blood and skin. To gain insight into abnormal gene expression promoting T cell dysfunction, lymphoproliferation and transformation in SS, we first compared functional transcriptomic profiles of both resting and activated CD4^+^CD45RO^+^ T cells from SS patients and normal donors to identified differential expressed genes. Next, a meta-analysis was performed to compare our SS data to public microarray data from a novel benign disease control, lymphocytic-variant hypereosinophilic syndrome (L-HES). L-HES is a rare, clonal lymphoproliferation of abnormal memory T cells that produces similar clinical symptoms as SS, including severe pruritus and eosinophilia. Comparison revealed gene sets specific for either SS (370 genes) or L-HES (519 genes), and a subset of 163 genes that were dysregulated in both SS and L-HES T cells compared to normal donor T cells. Genes confirmed by RT-qPCR included elevated expression of PLS3, *TWIST1* and *TOX* only in SS, while *IL17RB* mRNA was increased only in L-HES. *CDCA7* was increased in both diseases. In an L-HES patient who progressed to peripheral T cell lymphoma, the malignant transformation identified increases in the expression of *CDCA7*, *TIGIT*, and *TOX*, which are highly expressed in SS, suggesting that these genes contribute to neoplastic transformation. In summary, we have identified gene expression biomarkers that implicate a common transformative mechanism and others that are unique to differentiate SS from L-HES.

## INTRODUCTION

Sézary Syndrome (SS) is a rare and aggressive leukemic form of cutaneous T cell lymphoma (CTCL), characterized by pruritic erythroderma, lymphadenopathy, and leukemic T cells in the peripheral blood. The etiology of SS is unclear, but it is thought to develop from neoplastic transformation of mature CD4 T cells with Th2 bias and skin homing properties. SS can arise spontaneously, or develop in patients with a prior diagnosis of mycosis fungoides, a more skin-tropic and indolent form of CTCL. How microenvironmental factors influence the neoplastic T cell phenotype, and whether mycosis fungoides and SS have independent origins or represent a continuum of closely related T cell neoplasms remains unclear [1, 2]. A distinct clinical precursor of SS with CD4 lymphocytosis in blood and skin has not been identified, and gene expression studies often rely on benign dermatoses (e.g. chronic eczema, psoriasis and pityriasis rubra pilaris) for disease controls [3, 4]. Lymphocytic-variant hypereosinophilic syndrome (L-HES) also has clinical findings very similar to SS, and is derived from a benign lymphoproliferation of clonal T cells with skin and blood tropism. The persistent hypereosinophilia (AEC ≥1500 × 10^9^/L) of L-HES is secondary to IL-5 production by abnormal T cells [5–7], and both eosinophils and abnormal T cells are abundant in the blood and skin of L-HES patients [8–10]. Common dermatological manifestations of L-HES include pruritus, erythroderma, eczema, urticaria and angioderma [8–10]. These features make L-HES a valuable disease control for molecular studies of SS pathogenesis.

Unlike SS T cells, L-HES T cells lack cerebriform nuclei and appear largely normal, but SS and L-HES T cells share many molecular features. Like SS T cells, L-HES T cells are mature, memory T cells with a Th2 bias, and are often clonal [8, 11]. Absent or reduced expression of immunological surface markers associated with the T cell receptor complex (CD2, CD3, CD4, CD8) is common in both SS and L-HES T cells. In L-HES, the most common phenotypes are CD3^─^CD4^+^ and CD3^+^CD4^─^CD8^─^ [6]. Loss of CD7 is also common in both SS and L-HES T cells [8], while loss of CD26 appears limited to SS. L-HES typically has an indolent clinical course, and is considered a benign lymphoproliferative disorder. However, a small proportion of cases progress to full blown peripheral T cell lymphoma (PTCL) [10, 11]. Thus, SS and L-HES share a number of important features that suggest related developmental pathways.

As these T cell driven diseases share clinical and phenotypic similarities, we hypothesize that they also share a subset of similar gene expression abnormalities. Determining how gene expression in SS and L-HES differs from normal T cells and from each other may reveal genes that are potentially important to immune dysfunction, lymphoproliferation, neoplastic transformation, clinical symptoms, and unique biomarkers of each disease. Toward this purpose, we compared the functional transcriptomes of CD4^+^CD45RO^+^ T cells from SS patients and normal donors (ND) using high-density oligonucleotide microarrays. To gain new insight into genes important for SS and inflammation, we then conducted a meta-analysis of our own gene expression data for SS T cells and a public gene expression data set for L-HES T cells generated with the same microarray platform [12], and obtained from the Gene Expression Omnibus. Both studies examined inducible gene expression responses to assess functional differences. Meta-analysis of the two studies revealed both common and distinct gene expression patterns between SS and L-HES. Genes important as biomarkers to distinguish SS and/or L-HES were identified. In addition, examination of longitudinal data from one L-HES patient who progressed to T-lymphoma revealed that genes differentially expressed in SS were also dysregulated during this patient’s malignant clinical progression.

## RESULTS

### Functional alterations in transcriptomes of SS T cells compared to ND

**Figure 1:**
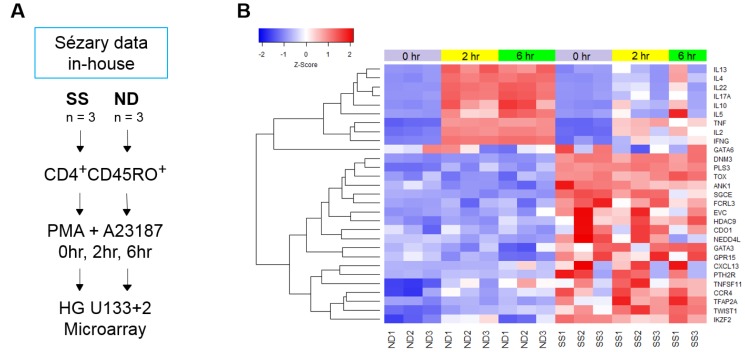
(**A**) Schematic of T cell populations and experimental design of the SS microarray study. CD4^+^CD45RO^+^ memory T cells were isolated from SS and ND PBMC by negative selection, as described in Materials and Methods. Cells from both SS and ND were activated with PMA+A23187 for 0, 2, and 6 hours. Gene expression was examined with Affymetrix HG U133 Plus 2.0 microarrays. (**B**) Traditional SS biomarker genes are highly over-expressed in SS compared to ND memory T cells, with little change in expression upon activation with PMA+A23187 in both SS and ND T cells. Stimulated cytokine gene expression is lower at 2 and 6 hours in SS compared to ND. Gene expression z-score is represented by a color scale from red (high expression) to blue (low expression). Colored bars at the top of the heat map indicate cell treatments: unstimulated (violet), 2 hour stimulated (yellow), and 6 hour stimulated (green).

**Figure 2:**
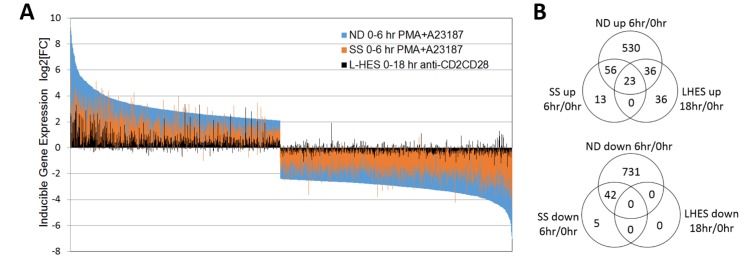
(**A**) Activation-dependent gene expression is globally reduced in SS compared to ND. All genes with significantly altered expression in ND are shown (blue bars), and ranked from highest to lowest relative expression vs unstimulated ND T cells. For the same genes, activation-dependent gene expression for SS is shown in orange, and L-HES in black. Gene expression log2FC in activated vs. resting T cells is represented by the direction of the bars from baseline, with increased expression above and decreased expression below zero. Each vertical bar represents an individual gene. ND (blue) and SS (orange) T cells were stimulated with PMA+A23187 for 0 or 6 hours. L-HES (black) T cells were stimulated with α-CD2CD28 and IL-2 for 0 or 18 hours. (**B**) Numbers of genes significantly up- or downregulated at the indicated time points are compared in separate Venn diagrams. Genes differentially expressed in stimulated vs. unstimulated T cells exceeded log2FC ≥ |1|, and pfp < 0.05. Supplementary Table 1 contains expression data for genes in each overlap category.

Analysis of T cell transcriptomes in response to stimulation offers functional insight and temporal regulation of gene expression not available from resting static T cells. We analyzed dynamic gene expression in resting and activated CD4^+^CD45RO^+^ memory T cells from three ND and three SS patients from cohort 1 (Figure 1A, Table 1, SS 1-3). Purified T cells were stimulated for 0, 2 and 6 hours with PMA+A23187, and mRNA expression was examined with high density Affymetrix HG U133 Plus 2 microarrays (Figure 1A). Comparing gene expression between resting CD4^+^CD45RO^+^ memory T cells from SS and ND identified 533 differentially expressed genes (DEGs, Supplementary Figure 1A), of which 190 DEGs (307 probes) were upregulated and 343 DEGs (536 probes) were downregulated in SS. The threshold for differential expression was log2 fold change (log2FC) ≥ |1| and percentage of false prediction (pfp) < 0.05, determined by the RankProduct method. Genes with significantly higher expression in SS compared to ND T cells included previously identified SS biomarker genes *CDO1, DNM3, GATA3, NEDD4L, PLS3, TOX*, and *TWIST1* (Figure 1B) [2, 4, 13–23]. These highly expressed genes can serve as biomarkers, and may have pathogenic roles [24, 25]. Deficiencies in *DPP4, SATB1*, and *STAT4* gene expression were also observed in our patients, recapitulating findings from prior studies. Reduced expression of *DPP4* mRNA is consistent with the CD26^─^ immunophenotype common to SS T cells. Biomarkers with reduced expression, such as *STAT4*, are most useful when combined with other positively expressed biomarker genes [3, 19]. Reduced expression of cytokine genes was also observed in stimulated SS T cells compared to ND (Figure 1B), confirming functional defects of SS T cells observed in our previous findings [26, 27]. Furthermore, a global deficit of functional, activation-dependent gene expression was apparent in SS T cells, with reduced amplitude of inducible gene expression compared to ND T cells (Figure 2A).

**Table 1:** Characteristics of Sézary syndrome patients in this study

Cohort 1	CTCL Stage	Gender	Race	Age years	Sézary cells %	Sézary cells/ul	
1^M^	IVA/SS	F	AA	75	45	4860	
2^M^	IVB/SS	M	W	64	29	5133	
3^M^	IVB/SS	F	W	53	61	6588	
4	IV/SS	M	W	62	63	5697	
5	IV/SS	F	AA	66	1	n.a.	
6	IV/SS	M	AA	61	18	n.a.	
7	IV/SS	F	W	92	0	n.a.	
8	IV/SS	F	W	70	16	n.a.	
9	IV/SS	F	AA	87	12	n.a.	
10	IV/SS	M	AA	66	n.a.	n.a.	
11	IV/SS	M	W	72	35	n.a.	
12	IV/SS	F	W	74	9	n.a.	
13	IV/SS	M	W	62	n.a.	n.a.	
**Cohort 2**	**CTCL Stage**	**Gender**	**Race**	**Age years**	**CD4:CD8 ratio**	**CD4+CD7− %**	**TCR clone +**
14	IVB/SS	M	AA	60	6.6	25	blood/skin
15	IVA/SS	M	W	71	47	45	n.d./skin
16	IVA/SS	M	AA	53	22	13	blood/skin
17	IVA/SS	M	AA	59	48	33	blood/n.d.
18	IVB/SS	F	W	63	30	31	blood/skin
19	IVB/SS	M	W	60	29	11	n.d./skin

^M^analyzed by microarray.

n.a. not available.

n.d. not determined.

**Figure 3:**
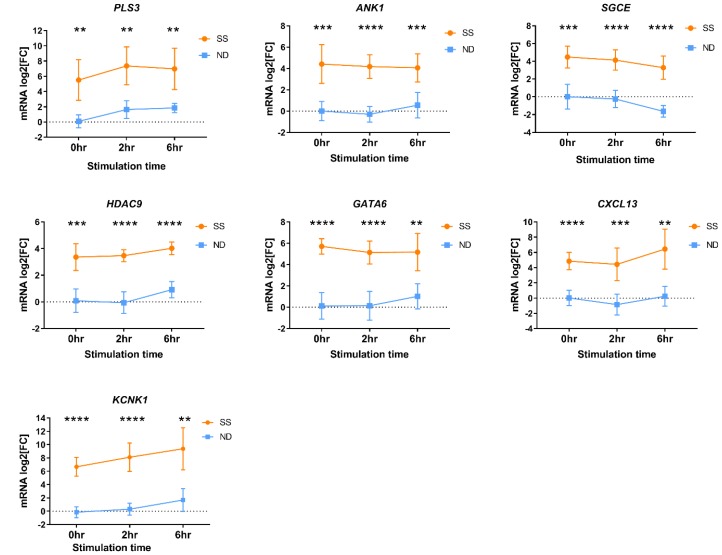
Differential gene expression measured by RT-qPCR in PBMCs from an independent group of SS patients (orange circles) and ND (blue squares) from cohort 1. PBMCs were stimulated with PMA+A23187 for 0, 2 and 6 hours. Differential gene expression is shown as the mean relative normalized mRNA level (mRNA log2FC) for 10-11 ND and 8-10 SS not represented by microarray data. Error bars represent 95% confidence intervals. ^*^ *P* < 0.05, ^**^ *P* < 0.01, ^***^ *P* < 0.001, ^***^ *P* < 0.0001 by 2-way ANOVA with Sidak’s post-test.

Differential expression of a subset of SS DEGs was validated by RT-qPCR (Figure 3) using PBMCs from an independent group of SS patients from cohort 1 (Table 1, SS 4-13). Significantly higher gene expression (*p* ≤ 0.05) was observed in PBMCs from SS patients compared to ND for *ANK1, CXCL13, KCNK1, GATA6, HDAC9, PLS3*, and *SGCE,* in resting PBMCs and following activation.

### Meta-analysis of SS and L-HES transcriptomes

**Figure 4:**
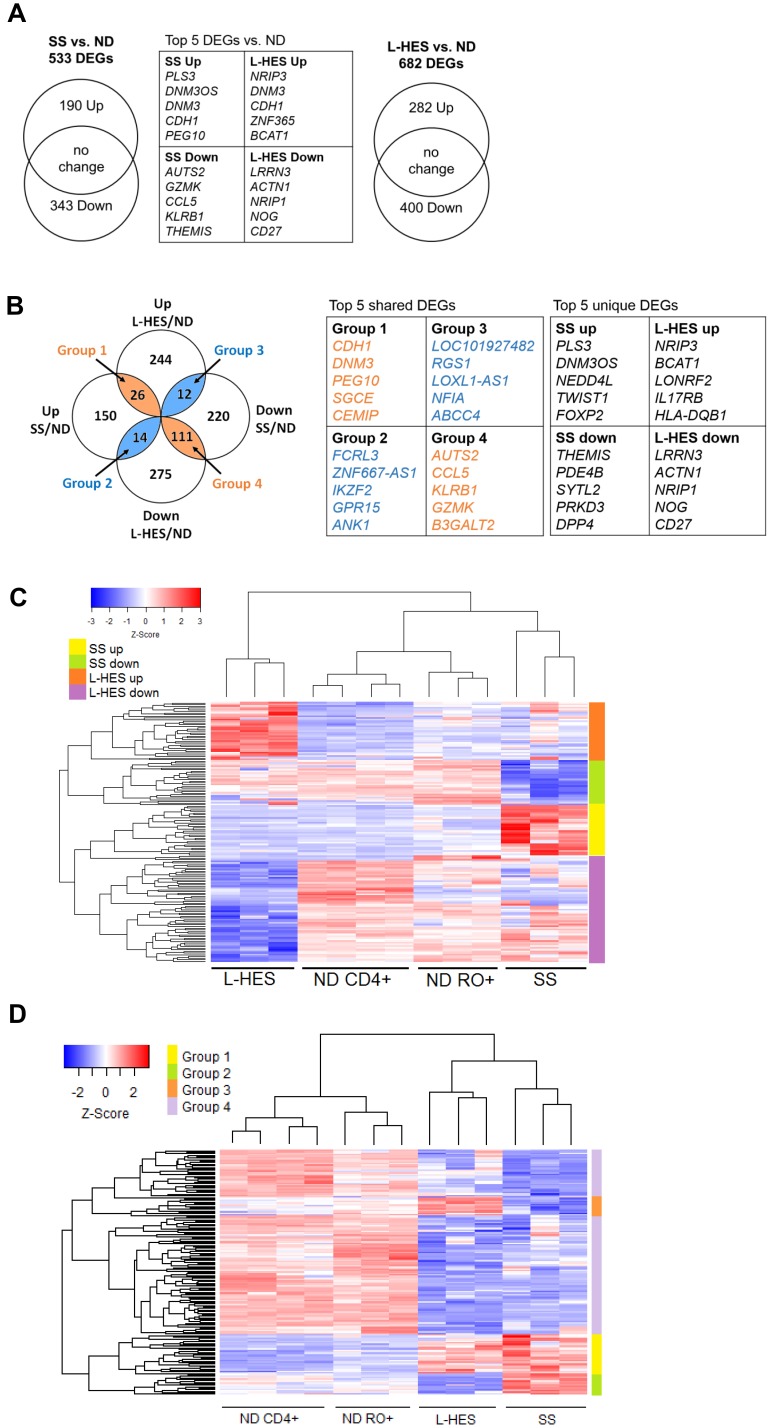
Meta-analysis of DEGs in SS and L-HES T cells. (**A**) Venn diagrams show the numbers of DEGs that were upregulated and downregulated in resting T cells from SS vs. ND, and L-HES vs. ND. (**B**) Groups of up- and downregulated DEGs for SS and L-HES from panel A were compared to each other using GeneVenn, and 163 shared DEGs were found. Concordantly changed DEGs are shown in orange overlap regions of the Venn diagram, and discordantly changed DEGs are shown in blue overlap regions. DEGs that were not shared between SS and L-HES are in the excluded white areas: 150 upregulated and 220 downregulated DEGs were unique to SS, and 247 upregulated and 276 downregulated DEGs were unique to L-HES. (**C**) Heatmap showing DEGs unique to SS or L-HES as distinct clusters. Genes with a 5-fold or greater mean change in gene expression are shown (Supplementary Tables 2 and 3). (**D**) Heat map showing four major groups of shared DEGs as distinct clusters (Supplementary Table 4). (**C**, **D**) Colored bars to the right of each heat map indicate groups of DEGs, as indicated by the color key in each panel. Gene expression is represented by a z-score color scale from red (high expression) to blue (low expression).

L-HES is a benign lymphoproliferation of T cells that exhibits both skin and blood infiltration, and Th2 bias like SS. L-HES and SS have common clinical features, but differ markedly in prognosis. We hypothesized that the clinical similarities may arise from similar gene expression profiles in the two T cell diseases, and comparing transcriptomic data for SS and L-HES T cells will provide insight into the pathogenesis of both diseases. A novel study by Ravoet, *et al*. [12] previously compared the transcriptomes of CD3^─^CD4^+^ T cells from three patients with chronic L-HES to CD3^+^CD4^+^ T cells from four ND (Table 2) using the HG U133 Plus 2.0 microarray platform and a similar experimental design as our SS study. In addition, the authors of this L-HES study demonstrated that the selected CD3^─^CD4^+^ T cells were overwhelmingly CD45RO^+^, like SS T cells. The public L-HES microarray data was obtained from the Gene Expression Omnibus, and reanalyzed using the same method as our data for SS (Supplementary Figure 2). This approach yielded 682 DEGs (1221 probes) in resting T cells from L-HES patients compared to ND (log2FC ≥ |1|, pfp < 0.05), of which 282 DEGs (496 probes) were upregulated and 400 DEGs (725 probes) were downregulated in L-HES (Figure 4A, Supplementary Figure 1B). Our analysis identified overexpression of *IL17RB*, *MAP3K8*, *RUNX2*, *SMAD5* and *TGFBR3*, and underexpression of *CYSLTR1*, *KIT*, *NOG*, *SMAD7*, *TGFBR1* and *TGFBR2* in L-HES T cells, as reported in the original study [12]. Significant overexpression of *GATA3* and *BATF* was also observed in resting L-HES T cells as reported previously [28].

**Table 2:** Summary of the L-HES microarray study

GEO accession number	GSE12079
Citation	Ravoet, *et al*. (2009)
L-HES T cells	*n* = 3, CD3^─^CD4^+^
ND T cells	*n* = 4, CD3^+^CD4^+^
Progression to T-lymphoma	Patient 1 only, year 6
Activation method	α-CD2CD28 + IL-2, 18 hours, L-HES only
Microarray platform	Affymetrix HG U133+2

A meta-analysis was then conducted to identify genes that were dysregulated either in SS or L-HES alone, or in both diseases. Comparing the 533 DEGs in SS to the 682 DEGs in L-HES (Figure 4A) revealed that many DEGs were unique to SS (150 up, 220 down) or unique to L-HES (244 up, 275 down), while 163 DEGs were shared between SS and L-HES (Figure 4B). Hierarchical clustering of a subset of SS-unique and L-HES-unique DEGs with 5 fold or greater differential expression separated SS from L-HES and produced four major clusters of up and downregulated genes for each disease (Figure 4C). The heatmap shows that a subset of DEGs significantly downregulated only in L-HES T cells (compared to bulk CD4 T cells) appear to also be somewhat reduced in SS and ND memory T cells. In contrast, other genes were uniquely downregulated in L-HES (compared to SS and all ND control T cells), including *IL17RB*, which may be an L-HES biomarker, as suggested previously [12]. DEGs unique to SS included many biomarker genes identified in prior studies, including *CDO1*, *PLS3*, *STAT4*, *TOX*, and *TWIST1* (Table 3) [3, 16, 19, 29]. The lack of altered expression for these genes in L-HES supports their potential to differentiate SS from L-HES.

**Table 3:** SS biomarker genes abnormally expressed in SS T cells, but not L-HES T cells

Gene Symbols	Sézary		L-HES		Sézary Citations
	log2FC	pfp	log2FC	pfp	
PLS3	7.04	1.21E-09	−0.34	8.64E-01	[13, 14, 16]
NEDD4L	3.93	7.77E-05	−0.72	2.51E-01	[16, 17]
TWIST1	3.81	1.07E-04	0.05	1.06E+00	[15–17]
PTH2R	3.18	3.28E-03	−0.16	1.10E+00	[21]
CDO1	3.13	9.80E-04	−0.71	1.87E-01	[16, 19, 21]
HDAC9	3.12	5.66E-04	−0.31	9.06E-01	[23, 68]
CXCL13	2.86	2.76E-03	0.11	1.06E+00	[69]
TOX	2.60	1.47E-03	0.28	1.02E+00	[4, 16, 17, 23]
GATA6	2.52	2.47E-03	−0.15	1.15E+00	[70, 71]
KLF8	2.24	3.34E-03	−0.82	2.41E-01	[17, 23]
TIGIT	1.60	1.59E-02	0.26	8.23E-01	[16, 23, 30]
STAT4	−3.35	9.72E-05	−0.37	7.88E-01	[13, 15, 17]
DPP4	−3.36	9.73E-05	0.32	6.99E-01	[13, 15]

The RankProd log2FC and pfp for each gene is presented for both diseases. For each gene, citations are provided for prior reports of differential gene expression in SS.

log2FC, log2 fold change.

pfp, percentage of false positives determined by RankProd.

The 163 DEGs shared between SS and L-HES included 135 (12.5%) that were concordantly altered (e.g., increased in both, or decreased in both; Figure 4B, orange overlap), and 26 (2%) that were discordantly altered (Figure 4B, blue overlap). The degree of concordant gene expression overlap is greater than expected by chance (*p* ≤ 1.027^−173^). Hierarchical clustering using all 163 shared DEGs for both SS and L-HES separated the four cell populations in the two studies (SS CD4^+^CD45RO^+^, ND CD4^+^CD45RO^+^, L-HES CD3^-^CD4^+^, ND CD3^+^CD4^+^) (Figure 4D). Heirarchical clustering also separated the four major groups of shared DEGs identified in Figure 4B (Figure 4D). Concordantly downregulated genes were separated into two clusters on the heat map, and will be collectively referred to as group 4 for the remainder of this report.

Each overlap group included genes with a previously published association with SS. Group 1 included genes overexpressed in both SS and L-HES: *CCR4* [2], CDCA7 [16, 17], *DNM3* [16, 19], *GATA3* [3, 13], *SGCE* [16, 21] and *TNFSF11* [15, 16]. Groups 2 and 3 showed discordant changes in SS and L-HES. *ANK1* [16, 18, 21] *FCRL3* [30, 31], and *IKZF2* [23, 30] were upregulated in SS and downregulated in L-HES (group 2), while *FAS* [32] was downregulated in SS and upregulated in L-HES (group 3). Group 4 included genes with decreased expression in both diseases: *BCL2L11* [15], *NKG7* [20], *GZMA* [17], *GZMK* [17, 20], *PLAC8* [17], *SATB1*, *SMAD7*, and *TGFBR2* [15, 17]. Group 4 also included several genes previously reported to be downregulated in L-HES, including *CYSLTR1*, *KIT*, *SMAD7*, *TGFBR1*, and *TGFBR2* [12]. DEGs shared by SS and L-HES may be involved in disease mechanisms common to both diseases, and cannot distinguish L-HES from SS.

The L-HES study used α-CD2CD28 and IL-2 (18 hours) for activation rather than the T cell receptor since CD3 was absent [12]. Only 95 genes were significantly upregulated, and no genes were significantly downregulated in activated vs. resting L-HES T cells (Figure 2B). In general, the largest changes in gene expression amplitude were observed in ND T cells, followed by SS T cells. L-HES showed the smallest changes following stimulation (Figure 2A). The L-HES study did not include α-CD2CD28-activated ND T cells, so it is unclear whether the lower activation of gene expression observed in L-HES result from suboptimal stimulation or intrinsic differences in cell phenotype, such as the loss of CD3 surface expression in L-HES T cells. In other studies, PMA+ionomycin and α-CD3CD28 beads yielded strikingly similar gene expression programs in T cells when examined with high density microarrays [33]. PMA+ionomycin and combinations of α-CD2CD28 or α-CD3CD28 also produce very similar increases in [^3^H]thymidine incorporation, but have more variable effects on chemokine production [34]. A recent study using PMA+ionomycin to activate L-HES T cells evoked larger increases in IL4, IL5 and IL13 mRNAs in L-HES T cells compared to control memory T cells [28]. This suggests that the lower induction of Th2 cytokine genes observed in the L-HES dataset is a result of the stimulation method, and not from an inability to activate these genes in L-HES T cells.

Due to the different activation methods used in the two studies, our analysis focused only on whether a gene met the significance threshold of pfp ≤ 0.05 for changes that exceeded 2-fold, and the direction of change, instead of comparing fold change amplitude. When lists of activated DEGs were compared between ND, SS, and L-HES, 23 genes were up-regulated in all three cell types (Figure 2B, Supplementary Table 1), including cytokines/chemokines (*IFNG*, *IL2*, *IL4*, *IL5*, *TNF*), transcription factors (*EGR1*, *EGR2*, *EGR3*, *IRF4*), and apoptosis/survival regulators (*PHLDA1*, *PMAIP1*, *SGK1*). In addition, 13 genes were significantly upregulated, and 5 genes were significantly downregulated only in SS (Figure 2B, Supplementary Table 1). *RND3, PIP5K1B*, and *SAMD5* increased by approximately 8 fold in SS, but remained unchanged in ND and L-HES (Supplementary Table 1). By comparison, 36 genes were significantly upregulated only in L-HES, including *BCL2,* *FLT3LG* and *IL17RB* (Figure 2B, Supplementary Table 1). Interestingly, *FLT3LG* was significantly downregulated in ND T cells.

**Figure 5:**
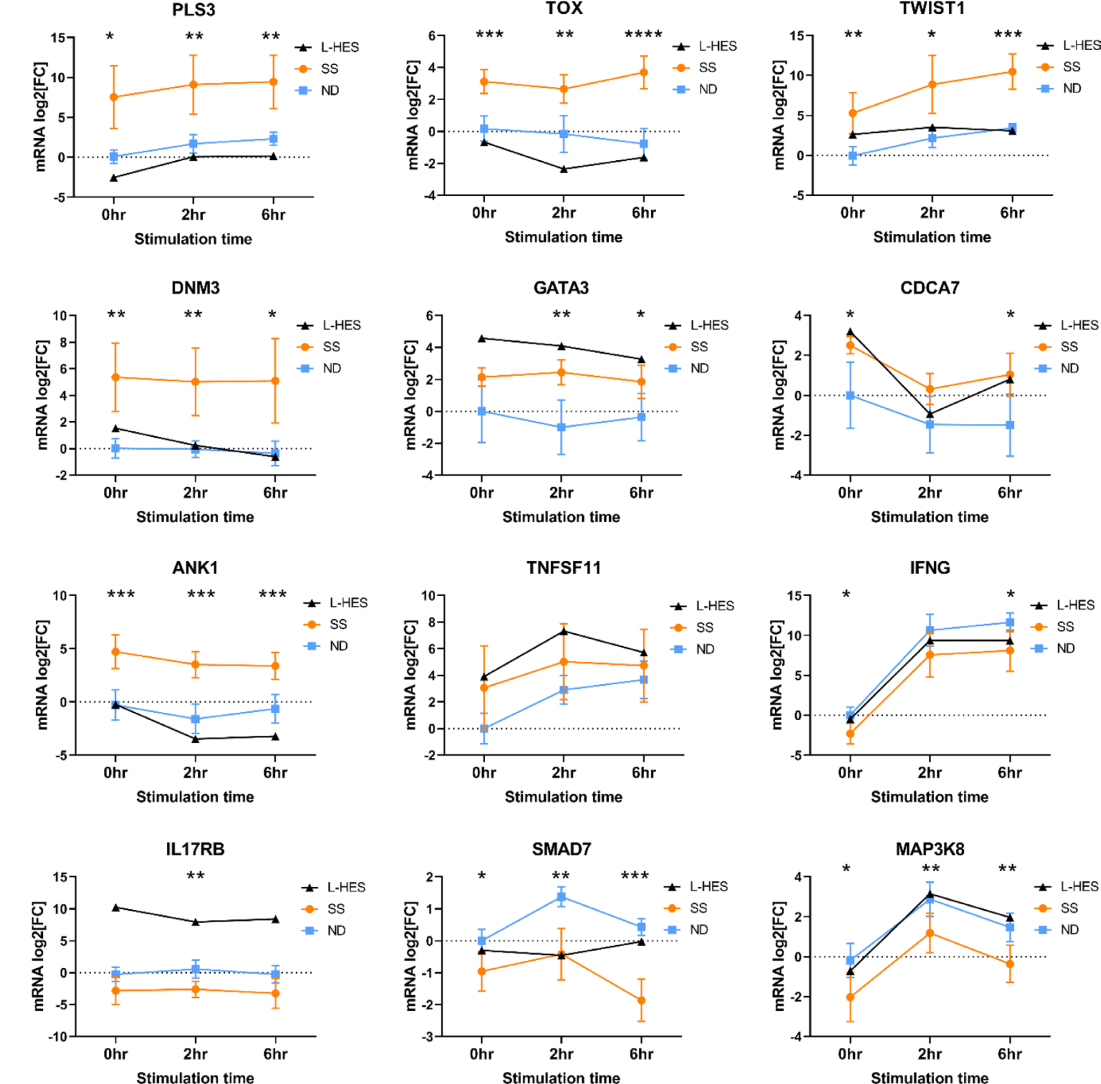
Validation of relative mRNA expression in SS and ND PBMCs from cohort 2, and an additional L-HES patient. PBMCs were stimulated with PMA+A23187 for 0, 2 and 6 hours. Differential gene expression is shown as the mean relative normalized mRNA level (mRNA log2FC) for 6 ND (blue squares) and 6 SS (orange circles) from cohort 2. L-HES data is from a single new patient. Error bars represent 95% confidence intervals. For SS vs. ND, ^*^ *P* < 0.05, ^**^ *P* < 0.01, ^***^ *P* < 0.001, ^***^ *P* < 0.0001 by 2-way ANOVA with Sidak’s post-test.

Gene expression changes were confirmed by RT-qPCR using PBMCs from an independent group of SS and ND from cohort 2 (Table 1, SS 14-19), and one additional L-HES patient (described in Materials and Methods, and summarized in Table 4). Results presented in Figure 5 support the concordant upregulation of the group 1 gene *CDCA7* in both SS and L-HES. The SS biomarker gene *DNM3* was only slightly elevated in this L-HES patient. Other group 1 genes *GATA3* and *TNFSF11* were upregulated in L-HES PBMCs but were not significantly elevated in SS. The group 2 gene *ANK1* showed significantly increased expression in SS PBMCs, and decreased expression was observed in the L-HES patient PBMCs following stimulation. The group 4 gene *SMAD7* showed significantly decreased expression in SS compared to ND, but decreased expression could not be confirmed in the L-HES patient PBMCs.

**Table 4:** Summary of L-HES patient characteristics for this study

Characteristic	Peripheral blood	Bone marrow
CD3─CD4+ cells	40%	23%
Absolute eosinophil count	2.6 × 10^9^/L	n.d.
Absolute lymphocyte count	12.6 × 10^9^/L	n.d.
Cytogenetic abnormalities	n.d.	normal
Gene rearrangements	n.d.	normal
TCRB clonality	n.d.	positive

n.d., not determined.

SS biomarker genes *PLS3*, *TOX* and *TWIST1* were not highly expressed in the L-HES microarray data compared to ND, and our RT-qPCR results from an independent L-HES patient corroborated low expression of *PLS3*, *TOX* and *TWIST1* (Figure 5). Microarray data also showed increased expression of *IL17RB* and *MAP3K8* in L-HES but not SS. *IL17RB* expression was not altered in SS PBMCs, but was elevated in L-HES PBMCs compared to ND (Figure 5). *MAP3K8* expression was significantly reduced in SS compared to ND, but the previously reported increased expression in L-HES could not be confirmed in PBMCs from this L-HES patient. Finally, *IFNG* expression was significantly reduced in SS PBMCs compared to ND, and results from the L-HES patient were intermediate at all time points.

### Biological process enrichment analysis

**Figure 6:**
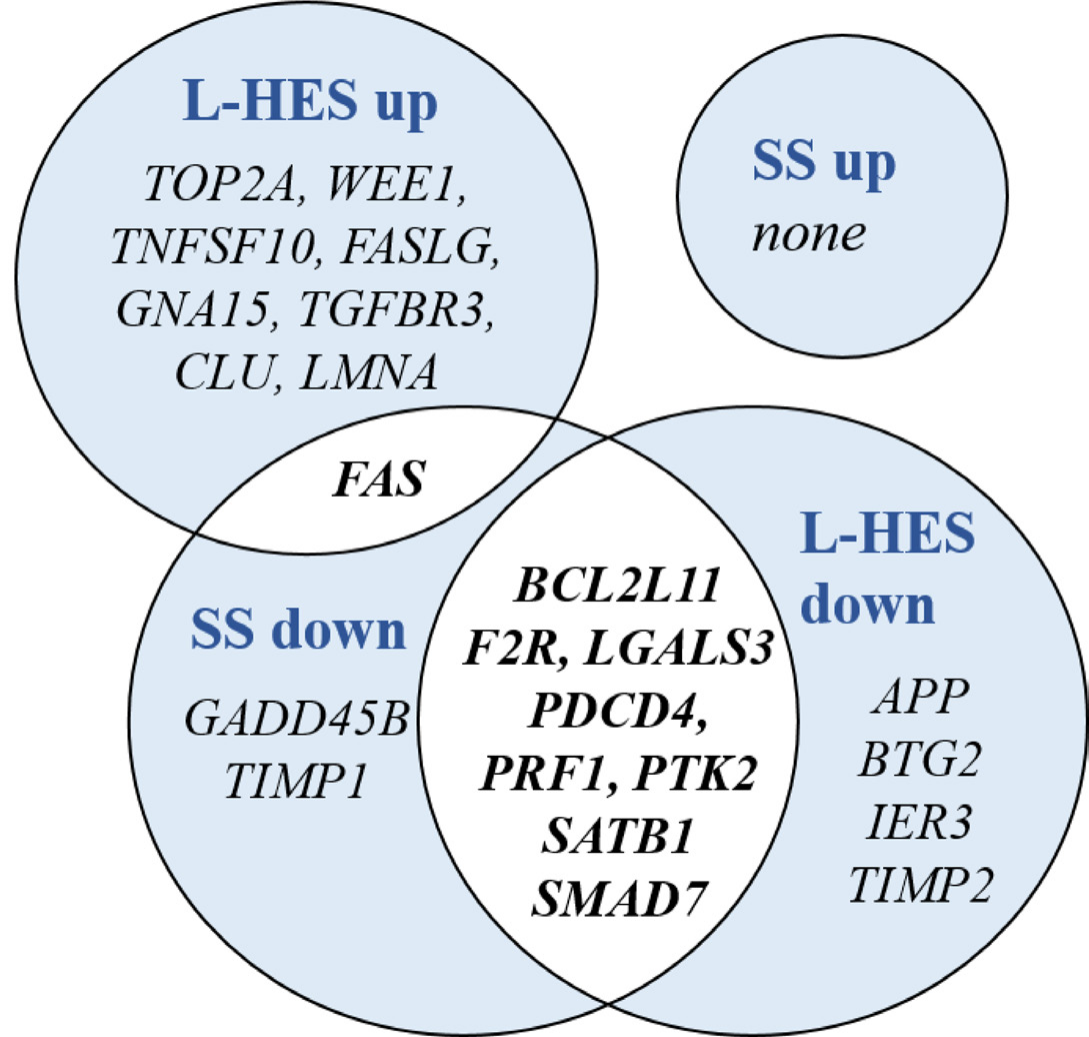
DEGs shared between SS and L-HES are enriched in the Hallmark gene set for apoptosis. Groups of up- and downregulated DEGs from SS and L-HES are depicted by overlapping circles. Gene symbols in overlapped areas with white background were dysregulated in both SS and L-HES, while gene symbols in areas with blue background were dysregulated only in SS or L-HES.

To reveal functional associations in the dysregulated genes of SS and L-HES, three gene lists for SS DEGs, L-HES DEGs, and shared DEGs were compared to annotated Hallmark gene sets from the Molecular Signatures Database (MSigDB) [35, 36], and overlapping genes were identified. The top ten Hallmark gene sets enriched by shared DEGs are presented in Table 5. The *IL-2 STAT5 signaling* and *Inflammatory Response* gene sets were enriched with both up- and down-regulated genes from both SS and L-HES DEG lists. However, all other gene sets lacked enrichment of up- or downregulated genes in one of the diseases. Five gene sets (*Apoptosis, Complement, Allograft Rejection, Interferon Gamma Response, and TNFα signaling via NF-κB*) were significantly enriched with both up- and downregulated genes in L-HES, but only downregulated genes in SS. Conversely, two Hallmark gene sets (*TGFβ Signaling, KRAS Signaling Up*) were significantly enriched with both up- and downregulated genes in SS, but only downregulated genes in L-HES. Figure 6 shows the genes that contribute to partially overlapping functional enrichment for the *Apoptosis* gene set. Except for the notable loss of *FAS* expression in SS, concordantly downregulated genes account for almost all of the overlap in this gene set. Additional non-shared DEGs unique to SS or L-HES suggest that apoptotic function is also altered in distinct ways in each disease.

**Table 5:** Overlap of SS DEGs, L-HES DEGs, and shared DEGs with MSigDB Hallmark gene sets

Hallmark biological process	Shared DEGs	SS DEGs		L-HES DEGs	
		Up	Down	Up	Down
IL-2 STAT5 Signaling	8.66E-13	5.39E-04	6.59E-19	5.13E-07	4E-14
Apoptosis	1.70E-07	n.s.	2.47E-07	3.78E-06	6.97E-06
Complement	7.78E-06	n.s.	3.76E-12	1.8E-02	1.46E-08
Inflammatory Response	7.78E-06	3.26E-02	3.34E-09	1.14E-04	1.46E-08
Allograft Rejection	6.39E-05	n.s.	4.07E-13	2.86E-06	2.59E-09
Interferon Gamma Response	6.39E-05	n.s.	3.34E-09	1.66E-05	2.59E-09
TGFβ Signaling	2.93E-04	7.58E-03	1.53E-05	n.s.	3.51E-03
KRAS Signaling Up	4.56E-04	7.76E-03	2.89E-08	n.s.	4.51E-03
TNFα Signaling via NF-κB	4.56E-04	n.s.	3.34E-09	1.66E-05	4.28E-11
IL-6 JAK STAT3 Signaling	1.32E-03	3.44E-03	1.01E-02	5.97E-05	n.s.

The top ten Hallmark gene sets that showed significant overlap with the 163 DEGs shared between SS and L-HES are listed. MSigDB overlap FDR *q*-values ≤ 0.05 are compared between groups of DEGs (n.s., not significant). SS DEG and L-HES DEG categories include all up- or downregulated genes exceeding log2FC ≥ |1| and RankProd pfp≤ 0.05.

### Malignant clinical progression of L-HES was associated with progressive change in SS genes

**Figure 7:**
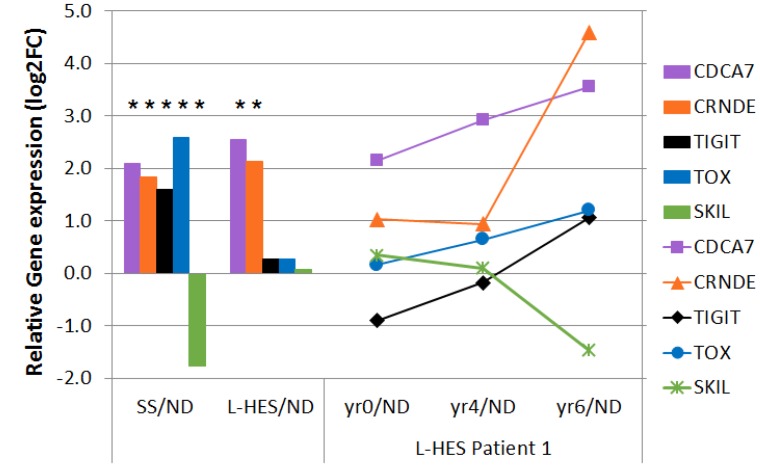
Disease progression in LP1 shows SS-like gene expression changes. Gene expression that changed more than 2 fold between year 0 (chronic L-HES) and year 6 (PTCL) in LP1 is shown for *CDCA7, CRNDE, TIGIT, TOX*, and *SKIL*. Line plots to the right show relative gene expression for LP1 years 0–6 compared to ND. Bar plots to the left show relative gene expression log2FC for all SS/ND and all L-HES(yr 0)/ND. Asterisks indicate significant results (pfp < 0.05, cases vs. controls).

L-HES patients are susceptible to progressing to peripheral T cell lymphoma (PTCL) (5–25% of cases), but detecting this transition can be challenging [10, 11]. The L-HES microarray study described one patient who developed malignant transformation from chronic L-HES in years 0–4 to an aggressive PTCL in year 6 of follow up [12]. We therefore determined if there were gene expression trends during the malignant clinical progression of L-HES patient 1 (LP1) that overlapped with SS gene expression. We compared all 533 SS DEGs (Figure 4A) to LP1 gene expression changes that exceeded 2-fold between years 0–4 (chronic L-HES) and year 6 (PTCL) of follow up. This yielded nine genes that increased and ten genes that decreased during LP1 progression, and mimmicked SS gene expression (Supplementary Table 5). Genes altered during LP1 progression in opposition to SS gene expression were excluded. *CDCA7* and *CRNDE* were over-expressed in both SS and L-HES by more than 2-fold between years 0-6. *CDCA7* increased progressively, while *CRNDE* increased only in year 6 (Figure 7). The SS biomarker genes *TIGIT* and *TOX* were overexpressed in SS, and increased progressively between years 0-6 during transformation to PTCL. The largest decrease was for *SKIL*, between years 4-6. *SKIL* was significantly downregulated in SS, but not in L-HES. In summary, a subset of genes were identified that changed during LP1 malignant progression to become more SS-like.

## DISCUSSION

**Figure 8:**
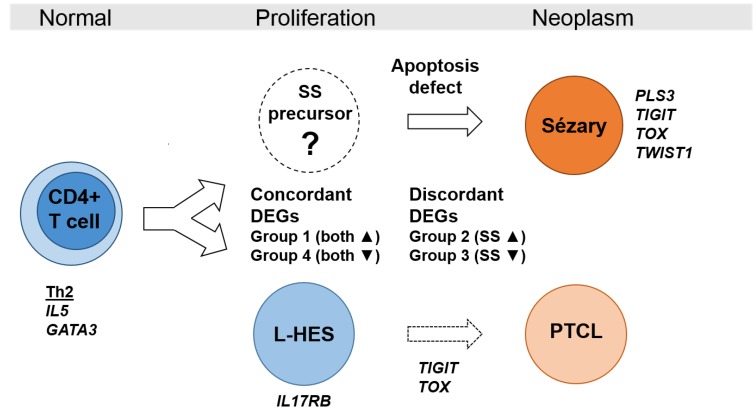
Model of gene expression in benign and neoplastic T cell lymhoproliferations. Clinical similarities and highly concordant gene expression in SS and L-HES suggest related etiologies. Concordant DEGs reflect the shared lymphocytosis and Th2 bias, while discordant DEGs and unique DEGs like *IL17RB* for L-HES and *PLS3* for SS highlight differences related to the neoplastic state in SS. Progression of chronic L-HES to PTCL was associated with increased expression of the SS genes *TIGIT* and *TOX*.

SS arises from the malignant transformation of skin homing memory T cells with Th2 bias, while L-HES is a benign lymphoproliferation of phenotypically similar T cells. We present the first comparison of differential gene expression in SS and L-HES to gain insight into these two similar diseases arising from memory T cells. From this novel approach, we identified (1) concordant gene expression that suggests related etiologies produce similarities in abnormal T cell phenotypes and clinical symptoms, (2) gene expression that is abnormal in both diseases but also discordant, and (3) gene expression unique to SS or L-HES (Figure 8). The discordant and unique DEGs may reflect differences important to malignancy, and can differentiate SS and L-HES. Finally, SS-like gene expression was observed to increase during progression of chronic L-HES to PTCL.

Higher expression of *ANK1*, *PLS3, TOX* and *TWIST1* in SS compared to ND and L-HES was confirmed in additional patients to support their value as biomarkers for SS. Overexpression of these genes may serve pro-oncogenic roles limited to SS and PTCL, such as apoptosis resistance attributed to PLS3 [37], and TOX-dependent repression of cyclin-dependent kinase inhibitors [25]. Several potential SS biomarker genes (*ANK1, CXCL13, GATA6, HDAC9, KCNK1, PLS3, SGCE, TWIST1*) maintained significantly higher expression in SS compared to ND in both resting and activated T cells, indicating that T cell activation would not diminish the ability of these genes to distinguish SS from ND. Gene expression unique to L-HES may also help distinguish these two clinically similar diseases. *IL17RB*, *MAP3K8, RUNX2, SMAD5* and *TGFBR3* were overexpressed only in L-HES, and increased *IL17RB* expression was confirmed in an L-HES patient in this study. Monitoring the expression of these genes may be helpful in excluding SS, and avoiding lymphoma therapies for L-HES patients.

A subset of 163 DEGs dysregulated in both SS and L-HES was also identified, and divided into four major groups based on the relationship of altered gene expression in the two diseases. Importantly, genes previously associated with SS were found to be concordantly dysregulated in both SS and L-HES, indicating that these genes are not specific biomarkers for neoplastic T cells in SS, and may have related functions in both diseases. The concordant overexpression of the “SS genes” *CCR4, DNM3, GATA3* and *TNFSF11* in both SS and L-HES (group 1) suggests common roles important to lymphocytosis, Th2 bias, and skin homing, and may be useful for classifying similar T-lymphoproliferations. *DNM3* has clinical implication in SS, as higher *DNM3* expression in 64 patients was associated with greater overall survival [19]. Elevated expression of *DNM3* in chronic L-HES is consistent with its proposed function as a tumor suppressor [38, 39]. The chemokine receptor CCR4 is essential for cutaneous homing of helper T cells associated with Th2-mediated pathology, and is the target of mogamulizumab, a monoclonal antibody clinically effective in SS (47% overall response rate) [40].

Group 1 also identified two genes with known pro-oncogenic roles without prior association with SS: *CDCA7*, and *CRNDE*. *CDCA7* overexpression has been linked to progression of chronic myelogenous leukemia to blast crisis [41], and its encoded protein, JPO1, is a direct target of c-myc [42]. *CRNDE* expression is up-regulated in many solid tumors and leukemias, and is associated with a stemness signature [43].

Concordantly downregulated genes in group 4 formed the largest category of shared DEGs in SS and L-HES. Many genes involved in Th1 (*IL18R1, IL18RAP, STAT4*), Th17 (*CCL20, CCR6, IL23A*), and effector (*EOMES, GZMA, GZMH, GZMK*) functions were underexpressed in both SS and L-HES T cells relative to ND T cells, reflecting Th2 lymphocytosis. Reduced *SATB1* expression may contribute to Th2 bias and proliferation in both SS and L-HES T cells. In CTCL cell lines, SATB1 represses *IL5* expression by displacing GATA3 from the *IL5* promoter [44]. Restoration of *SATB1* expression increased apoptosis in a SS cell line, suggesting that SATB1 deficiency may also promote apoptosis resistance in SS [17]. Thus, reduced *SATB1* expression in SS and L-HES may facilitate both eosinophilia and enhanced T cell proliferation.

Of the 163 genes that were differentially expressed in both SS and L-HES, 26 exhibited discordant differential expression. These included *FCRL3* and *ANK1*, which were high in SS and low in L-HES. Elevated *FCRL3* expression has been described in CD4^+^CD164^+^ T cells in SS [22, 31]. Increased *ANK1* expression in SS was validated for the first time in this study, and we confirmed that *ANK1* expression remained higher in SS compared to L-HES. Interestingly, *ANK1* harbors *miR-486*, which is overexpressed and involved in cell survival in SS [45]. Along with DEGs unique to SS or L-HES, discordant DEGs likely represent the divergent development of features such as malignancy.

Gene expression following cell activation was markedly different in SS, ND and L-HES. While we detected a core group of 23 genes that were significantly induced in all three cell types, the amplitude of inducible gene expression was globally reduced in SS compared to ND. Hampered inducible cytokine expression in SS was consistent with prior reports [26, 27], but the globally reduced amplitude of altered gene expression in SS in response to PMA+A23187 stimulation suggests that signaling defects in SS may not be limited to the T cell receptor complex. Activated L-HES T cells often produce more IL-5 than similarly activated normal T cells [11, 28]. Unfortunately, stimulation with α-CD2CD28 and IL-2 appeared to poorly activate L-HES T cells, as IL5 and other Th2 cytokines were not robustly activated, and this prevented useful comparisons outside of the most responsive genes.

Dysregulated gene expression in SS and L-HES was enriched with genes related to immune effector functions and apoptosis, including both upregulated and downregulated genes in L-HES, but only downregulated genes in SS. This is consistent with defects in immune function and apoptosis described for SS T cells [17, 46–48]. The *Apoptosis* gene set shared eight concordantly downregulated DEGs in SS and L-HES, including *BCL2L11, F2R, LGALS3, PDCD4, PRF1, PTK2, SATB1*, and *SMAD7*. *SMAD7* downregulation has been previously described in both SS and L-HES [12, 15], while down-regulation of *BCL2L11* and *SATB1* has been described only in SS [15, 17]. Discordant differential expression of *FAS* in SS and L-HES is consistent with their malignant and benign T cell phenotypes, respectively. In addition, the enrichment of upregulated genes in L-HES but not SS suggests that dysregulation of apoptotic pathways may also differentiate SS from L-HES.

Importantly, microarray data for the progression of L-HES patient LP1 from chronic L-HES to PTCL allowed assessment of genes with potential roles in malignancy. We assessed whether conversion of L-HES T cells to a malignant phenotype would be associated with the adoption of gene expression changes that resemble SS. The group 1 pro-oncogenes *CDCA7* and *CRNDE* were upregulated in both SS and L-HES, and both continued to rise during LP1 progression. Notably, the SS biomarker genes *TIGIT* and *TOX*, which were not significantly altered in chronic L-HES, increased at least two fold during LP1 progression. TIGIT is a co-inhibitory immunoreceptor, and SS patients with high TIGIT expression on CD4+ T cells also show high CD26 negativity [30]. *TOX* is one of the most studied biomarker genes for SS and CTCL [49–51], and high *TOX* transcript levels correlated with increased disease-specific mortality in SS [25].

In conclusion, comparison of SS and L-HES identified unique DEGs to differentiate SS from L-HES. Genes previously seen in SS such as *CCR4, GATA3*, and *TNFSF11*, but concordantly altered in SS and L-HES suggests common roles important in inflammation, lymphomagenesis and proliferation. *ANK1* and *CDCA7* are promising new biomarkers that have previously received little or no attention in SS. This is also the first time that increased expression of the SS biomarker genes *TIGIT* and *TOX* have been associated with clinical progression of chronic L-HES to PTCL, supporting their roles in driving malignancy. As the transcriptome studies were of limited sample size, continued studies with additional L-HES patients and comparisons to other Th2-driven diseases like atopic dermatitis may be helpful. Further studies of the functions of these genes in proliferation and malignancy will be important in understanding how these genes contribute to SS.

## MATERIALS AND METHODS

### Patients

L-HES patient characteristics, T cell enrichment, stimulation method, and microarray experiments were reported in detail by Ravoet, *et al*. [12], and are briefly summarized in Table 2. L-HES year 0 samples were used for all SS and L-HES comparisons, unless otherwise noted. L-HES patient 1 (LP1) was followed for 6 years, and samples from year 0 and year 4 represent chronic L-HES. LP1 was diagnosed in year 6 of follow-up with type 4 peripheral diffuse T lymphoma of small to medium lymphocytes [52].

Diagnosis of SS was based on World Health Organization-European Organization for Research and Treatment of Cancer staging and classification criteria [53]. All SS patients presented with an absolute Sézary cell count of at least 1,000 cells/mm^3^ at the time of diagnosis [54]. Two cohorts of patients diagnosed with SS were included in this study (Table 1). Cohort 1 was recruited between 2002–2005, under a research protocol approved by the Institutional Review Board of Henry Ford Hospital (Detroit, MI). The three patients represented in microarray experiments had a mean absolute Sézary cell count of 5,527 ± 929 cells/mm^3^. An independent group of 10 additional SS patients from cohort 1 was used for validation of SS gene expression in Figure 3. SS patients donated whole blood obtained by venipuncture. ND leucocytes were obtained from pheresis collars (*n* = 11, American Red Cross, Detroit, MI). Cohort 2 was recruited after January 2016, under a research protocol approved by the Institutional Review Board of the University of Arkansas for Medical Sciences (Little Rock, AR). PBMCs from six SS patients and one L-HES patient were used for the validation of gene expression in Figure 5. ND PBMCs were isolated from leucoreduction chambers (*n* = 6, Arkansas Blood Institute, Little Rock, AR). Diagnosis of L-HES was based on current criteria [11, 55]. This L-HES patient, a 73-year old black female, presented with generalized erythroderma and severe pruritis. The peripheral absolute cell counts were 2.6 × 10^9^/L for eosinophils and 12.6 × 10^9^/L for lymphocytes, and serum IgE was 3908 kU/L. Flow cytometry detected an abnormal population of CD3-CD4+ T cells in the peripheral blood (40% of total events) and bone marrow (23% of total events) that were heterogeneous for CD7. Bone marrow was positive for clonal T cell receptor gamma and beta rearrangements, but negative for cytogenetic abnormalities and gene rearrangements.

### Cells and treatments

Peripheral blood mononuclear cells (PBMC) were isolated from whole blood, pheresis collars, or leucoreduction chambers by density centrifugation using Ficoll [56]. We previously published Affimetrix HG U133 Plus2 microarray profiles of CD45RO^+^ (memory) and CD45RA^+^ (naïve) CD4^+^ T cells from healthy donors (*n* = 3 each) [26]. CD4^+^CD45RO^+^ T cells from SS patients (*n* = 3) described in the present report were isolated at the same time, and using the same methods, as described by Chong, *et al* [26]. Purified CD4^+^CD45RO^+^ T cells and PBMCs from SS and ND were stimulated with phorbol-12-myristate13-acetate (PMA) and A23187 ionophore (Calbiochem/Sigma), and then cultured for 2 and 6 hours, as described by Chong, *et al.* [26].

### Microarrays

Affymetrix HG U133 Plus 2 microarrays with 1.3 million probes covering 47 k transcripts were used. RNA purification, library preparation, and microarray hybridization was performed as described in Chong, *et al*. [26]. All activation time points (0, 2, 6 hours) are represented by three SS patients and three ND, except for the 6 hour time point, for which only two SS samples were available (SS1 and SS3, Figure 1). One microarray was used for each sample.

### RT-qPCR

Samples from 10-11 ND and 8-10 SS from Cohort 1 were used in Figure 3. Total RNA from PBMC was purified, reverse transcribed, and subjected to RT-qPCR as described previously [27]. Samples from 6 ND and 6 SS from Cohort 2 were used in Figure 5. Total RNA from PBMC was purified using RNeasy Plus kits (Qiagen), quantified by spectrophotometry at 260 nm and 280 nm, and reverse transcribed using VeriScript or Maxima kits (Life Technologies). RT-qPCR reactions were performed in triplicate using VeriQuest or Maxima SYBR Green qPCR master mixes (Life Technologies), on Applied Biosystems 7500 or QuantStudio 5 Real Time PCR Systems. β2-microglobulin mRNA expression was used for normalization of CT values. Primer sequences are provided in the Supplementary Table 6. Relative quantification of gene expression between SS and ND employed the 2^−ΔΔCT^ method [57]. Statistical analysis was performed with GraphPad Prism 8.0 software. For SS vs. ND, log2 transformed data was analyzed by 2-way repeated measures ANOVA with Sidak’s post test for multiple comparisons and alpha = 0.05. Gene expression for the single L-HES patient was considered differentially expressed if it differed by more than 2 standard deviations of the mean for ND or SS samples, depicted by 95% confidence intervals in Figure 5.

### Microarray data analysis

L-HES gene expression data based on Affimetrix HG U133 Plus 2 microarrays from Ravoet, *et al*. [12] was obtained from the Gene Expression Omnibus (https://www.ncbi.nlm.nih.gov/geo/) using accession number GSE12079. While several microarray studies for SS have been published [13, 16, 17, 58, 59], no other data set using the Affymetrix HG U133 Plus 2.0 platform is publicly available. The work flow for raw data processing, quality control measures, and analysis is summarized in the Supplementary Figure 2. Data analysis was performed in R version 3.4 (https://www.R-project.org/) using packages from Bioconductor (http://www.bioconductor.org/). Quality control checks on all Affymetrix CEL files from our laboratory and the downloaded CEL files were performed using arrayQualityMetrics [60]. One replicate of L-HES patient 3 (GSM304966) did not pass quality control and was excluded. CEL files that passed the quality control checks were background corrected and normalized using the frozen robust multiarray analysis (fRMA) [61]. fRMA performs well when microarray data is preprocessed individually or in small batches because quantile normalization is performed using a reference distribution created from a training database of 850 biologically diverse samples from public repositories. After normalization, probe-sets were assigned to Entrez genome annotations using the method from Dai, *et al.* [62]. ComBat [63] was used to correct a batch effect in the SS data set (Supplementary Figure 2) created by processing samples for two subjects (SS3, ND3) separately from the others. Control probes were filtered out from the expression data using geneFilter [64]. Ravoet, *et al*. [12] used single microarrays for ND samples and 2–3 microarray replicates for L-HES samples. We averaged replicate signal intensities prior to determining fold changes. Probes differentially expressed between cases and controls, or between stimulated and unstimulated conditions were identified using RankProd [65, 66]. The threshold for differential expression was log2FC ≥ |1|, and percentage of false prediction (pfp) < 0.05. Genes differentially expressed in both SS and L-HES were identified using GeneVenn [67]. Biological process enrichment was estimated using the Molecular Signatures Database (MSigDB) [36] hypergeometric overlap tool with the Hallmark gene set collection.

## SUPPLEMENTARY MATERIALS







## Abbreviations

CTCL: cutaneous T cell lymphoma
DEG: differentially expressed gene
L-HES: lymphocytic-variant hypereosinophilic syndrome
LP1: L-HES patient 1
ND: normal donor
PBMC: peripheral blood mononuclear cell
PTCL: peripheral T cell lymphoma
SS: Sézary syndrome
