# Supplementary material for: Transcriptome analysis of Sézary syndrome and lymphocytic-variant hypereosinophilic syndrome T cells reveals common and divergent genes
Source: Oncotarget. 2019 Aug 20;10(49):5052–69. doi: 10.18632/oncotarget.27120 (PMC6707948; doi:10.18632/oncotarget.27120)
Supplement: Supplementary file 1 [file oncotarget-10-5052-s001.pdf]

Transcriptome analysis of Sézary syndrome and lymphocytic-variant hypereosinophilic syndrome T cells reveals common and divergent genes

SUPPLEMENTARY MATERIALS

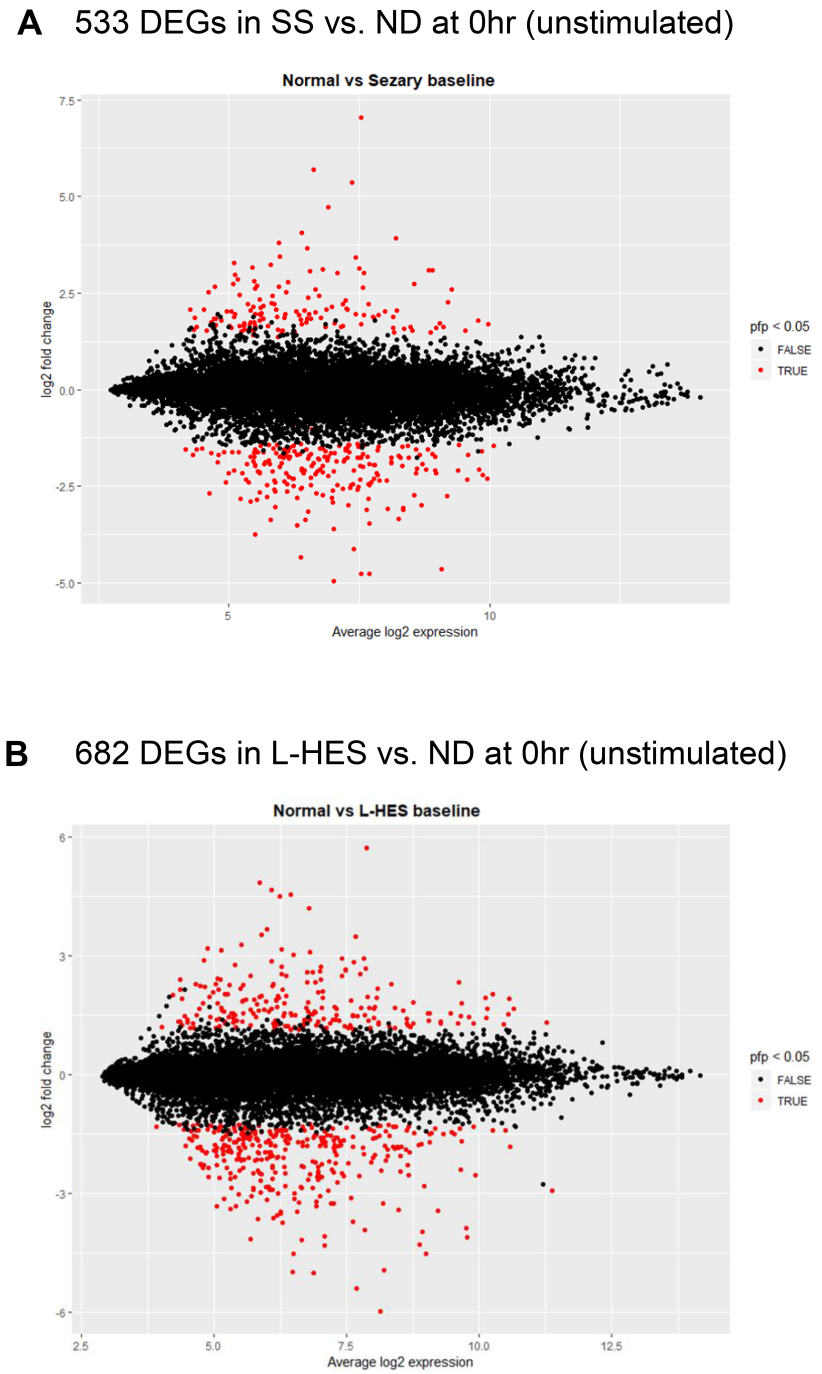

Supplementary Figure 1: MA Plots.

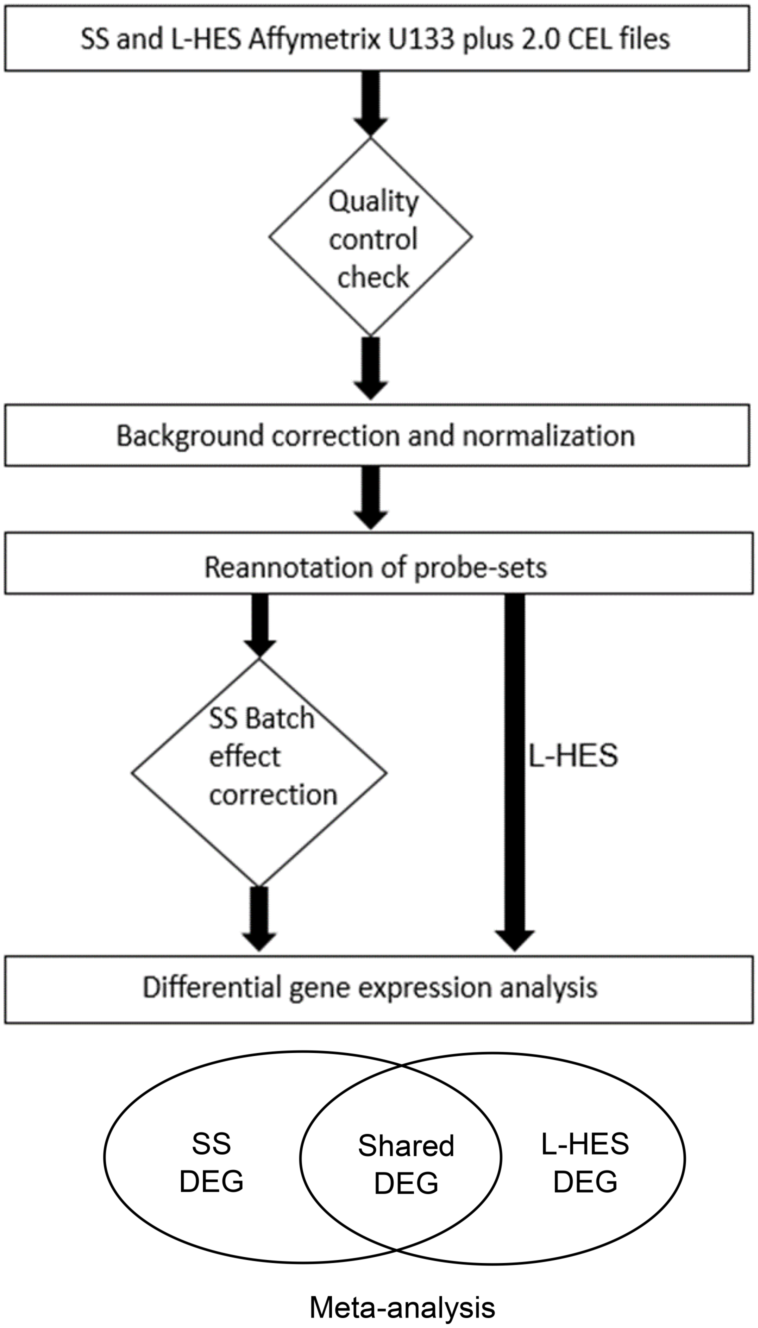

Supplementary Figure 2: Microarray data processing and meta-analysis.

Supplementary Table 1: Activated gene expression

				ND 0-6hr	ND 0-6hr	SS 0-6hr	SS 0-6hr	LHES 0-18hr	LHES 0-18hr
	Affy ID	Entrez_ ID	Symbols	log2FC	pfp	log2FC	pfp	log2FC	pfp
**All Up**	209795_at	969	*CD69*	3.36	6.1E-03	3.54	2.5E-02	1.14	4.4E-02
	209457_at	1847	*DUSP5*	4.85	4.3E-04	3.73	1.1E-02	2.55	8.2E-04
	227404_s_at	1958	*EGR1*	6.32	3.2E-05	4.17	7.5E-03	3.33	1.6E-04
	205249_at	1959	*EGR2*	6.48	2.5E-05	4.25	7.2E-03	2.69	7.9E-04
	206115_at	1960	*EGR3*	6.55	2.4E-05	4.66	4.2E-03	2.61	8.8E-04
	202345_s_at	2171	*FABP5*	4.38	1.0E-03	3.99	8.7E-03	1.86	4.5E-03
	210354_at	3458	*IFNG*	8.99	3.2E-08	3.23	4.9E-02	2.46	3.2E-03
	207849_at	3558	*IL2*	9.29	8.1E-09	5.40	3.0E-03	2.65	8.5E-04
	206341_at	3559	*IL2RA*	4.06	1.8E-03	4.00	6.7E-03	1.66	1.1E-02
	207538_at	3565	*IL4*	7.83	1.5E-06	3.29	2.0E-02	1.94	4.6E-03
	207952_at	3567	*IL5*	5.59	1.2E-04	3.17	1.1E-02	2.53	1.2E-03
	204562_at	3662	*IRF4*	6.30	3.6E-05	4.01	9.2E-03	2.55	7.6E-04
	224799_at	54602	*NDFIP2*	6.50	2.5E-05	3.22	2.9E-02	2.43	8.7E-04
	201577_at	4830	*NME1*	4.13	1.6E-03	2.88	5.0E-02	1.23	3.2E-02
	209959_at	8013	*NR4A3*	6.04	5.8E-05	3.72	1.0E-02	1.28	4.3E-02
	217996_at	22822	*PHLDA1*	4.12	1.6E-03	4.42	6.7E-03	1.44	2.6E-02
	204286_s_at	5366	*PMAIP1*	5.77	9.2E-05	3.16	3.0E-02	2.62	7.4E-04
	201739_at	6446	*SGK1*	4.01	2.0E-03	3.87	1.0E-02	2.03	3.1E-03
	201195_s_at	8140	*SLC7A5*	5.36	1.7E-04	2.94	9.2E-03	2.18	1.5E-03
	207113_s_at	7124	*TNF*	6.95	1.1E-05	3.55	1.4E-02	2.16	2.3E-03
	224553_s_at	8784	*TNFRSF18*	4.44	8.9E-04	3.12	2.9E-02	1.20	3.0E-02
	214228_x_at	7293	*TNFRSF4*	5.36	1.7E-04	3.96	9.4E-03	1.23	3.3E-02
	202241_at	10221	*TRIB1*	4.74	4.9E-04	3.06	4.5E-02	1.43	3.6E-02
**Only**	222108_at	347902	*AMIGO2*	1.68	1.0E-01	4.11	7.3E-03	–0.07	1.0E+00
**SS Up**	204637_at	1081	*CGA*	1.81	8.3E-02	3.27	2.9E-02	0.10	1.1E+00
	1554486_a_at	54438	*GFOD1*	1.26	2.0E-01	3.13	2.3E-02	–0.01	9.8E-01
	240063_at	441046	*GUSBP5*	1.96	6.4E-02	2.83	5.0E-02	0.28	9.5E-01
	219403_s_at	10855	*HPSE*	0.59	9.9E-01	2.86	2.9E-02	0.46	6.5E-01
	226189_at	3696	*ITGB8*	0.77	6.3E-01	3.02	3.5E-02	–0.09	1.0E+00
	206637_at	9934	*P2RY14*	–0.44	9.8E-01	2.98	1.6E-02	0.54	4.4E-01
	205632_s_at	8395	*PIP5K1B*	–0.83	5.2E-01	3.30	9.0E-03	0.20	1.1E+00
	202988_s_at	5996	*RGS1*	1.75	1.3E-01	3.49	1.0E-02	1.13	1.0E-01
	212724_at	390	*RND3*	0.27	1.2E+00	3.39	1.7E-02	0.20	1.1E+00
	228653_at	389432	*SAMD5*	–0.01	1.3E+00	2.94	3.9E-02	0.04	1.0E+00
	230650_at	81796	*SLCO5A1*	0.14	1.1E+00	3.31	2.4E-02	0.00	1.0E+00
	228715_at	170261	*ZCCHC12*	0.85	4.2E-01	2.73	4.7E-02	–0.22	1.0E+00
**Only**	229725_at	23305	*ACSL6*	–1.15	3.9E-01	–3.23	3.6E-02	–0.13	9.4E-01
**SS Down**	1555989_at	23002	*DAAM1*	–0.70	6.5E-01	–2.11	3.8E-02	–0.58	6.9E-01
	226974_at	23327	*NEDD4L*	–0.91	4.7E-01	–4.06	7.6E-03	0.01	1.0E+00
	213093_at	5578	*PRKCA*	–1.73	1.5E-01	–3.03	3.7E-02	–0.40	8.6E-01
	208998_at	7351	*UCP2*	–1.59	1.9E-01	–3.35	3.3E-02	0.09	1.1E+00

**Only**	221648_s_at	79814	*AGMAT*	0.87	4.8E-01	0.89	4.0E-01	1.20	4.6E-02
**L-HES Up**	1557236_at	80830	*APOL6*	1.41	1.6E-01	0.53	9.3E-01	1.43	4.9E-02
	203685_at	596	*BCL2*	–1.00	4.5E-01	–0.04	1.1E+00	3.06	5.5E-04
	210108_at	776	*CACNA1D*	0.01	1.1E+00	0.36	9.0E-01	1.91	2.6E-02
	209619_at	972	*CD74*	–0.37	1.1E+00	0.55	1.0E+00	1.60	8.3E-03
	1554519_at	941	*CD80*	1.02	3.9E-01	0.12	1.1E+00	1.74	2.5E-02
	205101_at	4261	*CIITA*	0.20	1.2E+00	0.11	1.1E+00	1.57	7.7E-03
	226702_at	129607	*CMPK2*	–1.38	2.8E-01	–0.98	4.0E-01	1.93	3.7E-03
	231776_at	8320	*EOMES*	0.67	7.3E-01	0.25	1.1E+00	1.76	3.6E-02
	231926_at	58513	*EPS15L1*	1.84	7.5E-02	1.08	4.6E-01	1.21	4.9E-02
	210607_at	2323	*FLT3LG*	–2.61	3.5E-02	–0.60	8.5E-01	1.91	3.1E-03
	203853_s_at	9846	*GAB2*	–0.71	6.4E-01	–0.62	5.4E-01	1.33	3.3E-02
	235175_at	115361	*GBP4*	1.57	1.2E-01	0.95	4.1E-01	1.65	1.4E-02
	213069_at	57493	*HEG1*	0.77	5.4E-01	0.93	4.9E-01	1.20	3.6E-02
	236203_at	3117	*HLA-DQA1*	0.45	1.0E+00	–0.06	1.2E+00	1.47	4.4E-02
	208894_at	3122	*HLA-DRA*	–1.13	3.6E-01	–0.20	1.1E+00	2.00	6.3E-03
	221170_at	59340	*HRH4*	1.09	3.4E-01	1.24	4.2E-01	1.48	3.0E-02
	229450_at	3437	*IFIT3*	1.14	3.6E-01	0.73	7.9E-01	1.78	1.5E-02
	219255_x_at	55540	*IL17RB*	–0.02	1.3E+00	0.40	1.1E-00	1.52	7.5E-03
	208949_s_at	3958	*LGALS3*	–2.20	7.4E-02	–1.45	1.6E-01	1.73	6.1E-03
	220121_at	55180	*LINS1*	2.10	5.1E-02	–0.08	1.2E+00	1.36	2.4E-02
	229327_s_at	4094	*MAF*	1.44	1.6E-01	1.98	1.6E-01	1.52	2.4E-02
	225662_at	51776	*MAP3K20*	–0.13	1.4E+00	0.25	1.1E+00	2.04	3.3E-03
	203414_at	23531	*MMD*	–0.20	1.1E+00	0.68	5.0E-01	1.38	1.8E-02
	218051_s_at	64943	*NT5DC2*	0.54	9.0E-01	0.19	1.1E+00	1.16	4.4E-02
	223220_s_at	83666	*PARP9*	0.07	1.2E+00	–0.35	8.6E-01	1.36	2.2E-02
	213469_at	80055	*PGAP1*	1.57	1.2E-01	1.06	3.2E-01	1.55	1.2E-02
	209193_at	5292	*PIM1*	0.42	1.0E+00	–0.36	9.2E-01	1.18	3.4E-02
	206631_at	5732	*PTGER2*	0.89	5.1E-01	–0.60	7.5E-01	2.08	1.6E-02
	230230_at	5775	*PTPN4*	–2.03	9.6E-02	–0.75	6.6E-01	1.12	4.7E-02
	204040_at	9781	*RNF144A*	–2.12	8.1E-02	–1.14	5.1E-01	1.24	3.2E-02
	242625_at	91543	*RSAD2*	–0.02	9.7E-01	–0.40	9.1E-01	1.92	3.2E-03
	202375_at	9871	*SEC24D*	–0.15	1.4E+00	–0.13	1.2E+00	1.16	4.6E-02
	231628_s_at	5269	*SERPINB6*	0.07	1.1E+00	–0.05	1.1E+00	4.49	1.1E-05
	217979_at	27075	*TSPAN13*	2.15	5.2E-02	1.22	2.4E-01	2.33	3.3E-03

Nonsignificant results are in gray text.

Supplementary Table 2: Genes and probes differentially expressed (0 hr) in SS vs. ND, but not in L-HES vs. ND

			SS/ND	SS/ND	L-HES/ND	L-HES/ND
**Affymetrix ID**	**Entrez_IDs**	**Symbols**	**log2FC**	**pfp**	**log2FC**	**pfp**
201215_at	5358	*PLS3*	7.04	1.21E-09	–0.34	8.64E-01
232090_at	100628315	*DNM3OS*	5.69	6.57E-07	0.13	1.06E+00
226974_at	23327	*NEDD4L*	3.93	7.77E-05	–0.66	2.93E-01
213943_at	7291	*TWIST1*	3.81	1.07E-04	0.05	1.06E+00
235891_at	100628315	*DNM3OS*	3.80	1.22E-04	0.10	1.06E+00
212445_s_at	23327	*NEDD4L*	3.52	2.07E-04	–0.73	2.51E-01
212448_at	23327	*NEDD4L*	3.43	2.97E-04	–0.31	9.64E-01
235201_at	93986	*FOXP2*	3.33	4.18E-04	0.39	8.05E-01
243278_at	93986	*FOXP2*	3.28	5.19E-04	0.06	1.05E+00
206772_at	5746	*PTH2R*	3.18	3.28E-03	–0.16	1.10E+00
204154_at	1036	*CDO1*	3.13	9.80E-04	–0.71	1.87E-01
205659_at	9734	*HDAC9*	3.12	5.66E-04	–0.31	9.06E-01
205159_at	1439	*CSF2RB*	3.10	5.75E-04	–1.39	5.22E-02
235171_at	100505501	*LOC100505501*	3.01	5.86E-04	0.49	4.90E-01
219654_at	9200	*HACD1*	3.01	3.34E-03	–0.91	1.12E-01
230550_at	64231	*MS4A6A*	2.98	2.44E-03	–0.64	2.51E-01
229281_at	64067	*NPAS3*	2.98	9.49E-04	–0.04	1.11E+00
205242_at	10563	*CXCL13*	2.86	2.76E-03	0.11	1.06E+00
203962_s_at	10529	*NEBL*	2.81	5.92E-03	–0.09	1.15E+00
1555867_at	2786	*GNG4*	2.74	5.75E-03	0.09	1.05E+00
203961_at	10529	*NEBL*	2.73	2.56E-03	0.05	1.05E+00
201939_at	10769	*PLK2*	2.70	1.28E-03	0.10	1.05E+00
204451_at	8321	*FZD1*	2.67	1.54E-03	–0.40	7.57E-01
206007_at	10216	*PRG4*	2.66	1.35E-03	0.11	1.05E+00
227195_at	84858	*ZNF503*	2.62	1.43E-03	–0.13	1.14E+00
204529_s_at	9760	*TOX*	2.60	1.47E-03	0.28	1.02E+00
219666_at	64231	*MS4A6A*	2.59	6.15E-03	–0.39	6.92E-01
230412_at	64067	*NPAS3*	2.57	1.92E-03	0.24	9.62E-01
230551_at	283455	*KSR2*	2.53	1.80E-03	–0.21	1.10E+00
210002_at	2627	*GATA6*	2.52	2.47E-03	–0.15	1.15E+00
224356_x_at	64231	*MS4A6A*	2.51	1.14E-02	–0.23	1.12E+00
225665_at	51776	*MAP3K20*	2.46	1.95E-03	0.38	6.33E-01
223280_x_at	64231	*MS4A6A*	2.45	9.59E-03	–0.72	2.02E-01
204653_at	7020	*TFAP2A*	2.43	2.78E-03	0.77	1.82E-01
205184_at	2786	*GNG4*	2.43	3.34E-03	0.34	9.17E-01
208179_x_at	3804	*KIR2DL3*	2.43	3.35E-03	–0.06	1.14E+00
230109_at	27115	*PDE7B*	2.39	4.41E-03	–0.78	2.31E-01
238447_at	27303	*RBMS3*	2.37	4.97E-03	–0.47	5.08E-01
232195_at	57512	*GPR158*	2.34	5.76E-03	0.23	1.05E+00
230968_at	9734	*HDAC9*	2.33	3.47E-03	0.11	1.06E+00
226705_at	2260	*FGFR1*	2.33	3.02E-03	–0.28	9.52E-01

223434_at	2635	*GBP3*	–2.35	1.82E-03	–0.67	3.88E-01
203716_s_at	1803	*DPP4*	–2.36	1.39E-03	0.20	1.02E+00
205005_s_at	9397	*NMT2*	–2.39	1.50E-03	–0.38	7.06E-01
206676_at	1088	*CEACAM8*	–2.40	1.55E-03	–0.07	1.13E+00
212993_at	138151	*NACC2*	–2.41	1.24E-03	–0.65	2.94E-01
226425_at	79745	*CLIP4*	–2.45	1.34E-03	0.02	1.05E+00
205006_s_at	9397	*NMT2*	–2.45	1.20E-03	–0.56	4.69E-01
223681_s_at	10207	*PATJ*	–2.45	1.04E-03	0.18	1.04E+00
209163_at	1534	*CYB561*	–2.46	1.04E-03	0.42	8.09E-01
223887_at	29933	*GPR132*	–2.49	9.49E-04	0.51	6.80E-01
206978_at	729230	*CCR2*	–2.55	7.99E-04	1.22	8.79E-02
206991_s_at	1234	*CCR5*	–2.55	8.32E-04	–0.28	9.04E-01
202018_s_at	4057	*LTF*	–2.56	8.05E-04	0.41	7.52E-01
212526_at	23111	*SPART*	–2.58	9.62E-04	–1.16	5.91E-02
223809_at	64407	*RGS18*	–2.63	6.99E-04	0.96	3.25E-01
1555370_a_at	23261	*CAMTA1*	–2.65	6.09E-04	–0.92	2.58E-01
201060_x_at	2040	*STOM*	–2.66	5.97E-04	–1.04	1.24E-01
221916_at	4747	*NEFL*	–2.68	6.06E-04	0.95	9.29E-02
212730_at	23336	*SYNM*	–2.69	6.00E-04	0.44	4.42E-01
206363_at	4094	*MAF*	–2.71	5.84E-04	–0.96	1.91E-01
224367_at	84707	*BEX2*	–2.73	5.49E-04	0.08	1.05E+00
217963_s_at	27018	*BEX3*	–2.77	4.74E-04	–0.33	7.75E-01
229327_s_at	4094	*MAF*	–2.77	4.66E-04	–0.85	3.15E-01
1558739_at	118426	*BORCS5*	–2.80	4.83E-04	–0.92	1.91E-01
221805_at	4747	*NEFL*	–2.86	4.13E-04	0.99	5.82E-02
222108_at	347902	*AMIGO2*	–2.92	3.50E-04	–0.13	1.14E+00
228532_at	128346	*C1orf162*	–2.99	2.91E-04	1.02	5.87E-02
203717_at	1803	*DPP4*	–2.99	2.96E-04	0.19	1.02E+00
225496_s_at	54843	*SYTL2*	–3.06	2.61E-04	–0.95	1.48E-01
201061_s_at	2040	*STOM*	–3.07	2.49E-04	–1.19	1.04E-01
209348_s_at	4094	*MAF*	–3.08	2.49E-04	–0.64	3.36E-01
214146_s_at	5473	*PPBP*	–3.12	4.10E-04	–0.55	3.79E-01
206118_at	6775	*STAT4*	–3.35	9.72E-05	–0.37	7.88E-01
211478_s_at	1803	*DPP4*	–3.36	9.73E-05	0.32	6.99E-01
218236_s_at	23683	*PRKD3*	–3.37	9.67E-05	–0.41	5.86E-01
211302_s_at	5142	*PDE4B*	–3.44	8.34E-05	–0.78	2.40E-01
232914_s_at	54843	*SYTL2*	–3.52	5.89E-05	–1.01	1.23E-01
1558971_at	387357	*THEMIS*	–3.91	1.78E-05	0.75	3.20E-01
203708_at	5142	*PDE4B*	–4.13	5.22E-06	–0.83	2.12E-01
1558972_s_at	387357	*THEMIS*	–4.35	3.07E-06	0.82	1.58E–01

This table corresponds to Figure 4C, and includes probes with differential expression of log2FC ≥ |2.33| (or 5 fold) and percentage of false prediction (pfp) < 0.05 in SS, as determined by the RankProduct method. Nonsignificant results are in gray text.

Supplementary Table 3: Genes and probes differentially expressed (0 hr) in L-HES vs. ND, but not in SS vs. ND. See Supplementary Table 3

Supplementary Table 4: Genes and probes differentially expressed in both SS and LHES (0 hr). See Supplementary Table 4

Supplementary Table 5: Genes and probes that acquired SS-like changes during LP1 progression

			**LP1 yr0/ND**	**LP1 yr4/yr0**	**LP1 yr6/yr0**	**SS/ND 0 hr**	**SS/ND 0 hr**	**L-HES/ ND 0 hr**	**L-HES/ND 0 hr**
**Affymetrix ID**	**Entrez_IDs**	**Symbols**	**log2FC**	**log2FC**	**log2FC**	**log2FC**	**pfp**	**log2FC**	**pfp**
212736_at	89927	C16orf45	−0.01	0.08	**1.01**	**1.30**	3.4E-02	0.20	1.0E+00
224428_s_at	83879	CDCA7	2.15	0.77	**1.40**	**2.09**	5.7E-03	2.55	9.5E-04
238021_s_at	643911	CRNDE	1.03	−0.09	**3.55**	**1.83**	1.8E-02	2.14	4.3E-03
205898_at	1524	CX3CR1	−2.23	−0.04	**1.00**	**2.65**	1.9E-03	−1.72	1.3E-02
201508_at	3487	IGFBP4	0.13	0.29	**1.65**	**1.79**	4.7E-02	0.23	1.0E+00
227725_at	55808	ST6GALNAC1	0.07	0.52	**2.35**	**1.61**	2.6E-02	0.87	1.8E-01
223529_at	6860	SYT4	−0.19	0.11	**1.02**	**1.42**	2.5E-02	0.94	1.6E-01
240070_at	201633	TIGIT	−0.90	0.72	**1.96**	**1.60**	1.6E-02	0.26	8.2E-01
204529_s_at	9760	TOX	0.16	0.48	**1.04**	**2.60**	1.5E-03	0.28	1.0E+00
201849_at	664	BNIP3	−1.51	−0.85	**−1.03**	**−1.21**	4.8E-02	−2.01	6.8E-03
239072_at	133418	EMB	0.96	−1.07	**−1.14**	**−1.54**	1.9E-02	0.79	3.5E-01
206404_at	2254	FGF9	−0.87	−0.53	**−1.07**	**−2.38**	1.3E-03	−1.77	1.5E-02
225792_at	51361	HOOK1	−1.60	−0.69	**−1.33**	**−1.71**	1.5E-02	−2.90	8.2E-04
210095_s_at	3486	IGFBP3	−0.27	−0.28	**−1.25**	**−2.15**	2.3E-03	−1.55	4.3E-02
221805_at	4747	NEFL	1.29	−0.82	**−1.75**	**−2.86**	4.1E-04	0.99	5.8E-02
221916_at	4747	NEFL	1.22	−0.80	**−1.78**	**−2.68**	6.1E-04	0.95	9.3E-02
205259_at	4306	NR3C2	−1.90	−0.44	**−1.06**	**−1.76**	1.9E-02	−1.36	7.0E-02
217865_at	55819	RNF130	−0.65	−0.40	**−1.07**	**−1.98**	4.6E-03	−1.60	2.5E-02
225227_at	6498	SKIL	−0.69	0.04	**−1.02**	**−1.80**	7.8E-03	−0.54	4.0E-01
217591_at	6498	SKIL	0.35	−0.25	**−1.82**	**−1.78**	1.1E-02	0.08	5.3E-01
213994_s_at	10418	SPON1	−1.79	−0.34	**−1.13**	**−1.92**	1.4E-02	−2.72	1.1E-03

Nonsignificant results are in gray text.
LP1 yr0/ND, LP1 year 0 gene expression compared to ND mean at year 0.
LP1 yr4/yr0, LP1 year 4 gene expression compared to LP1 year 0.
LP1 yr6/yr0, LP1 year 6 gene expression compared to LP1 year 0.
SS/ND, SS mean compared to ND mean, resting (0 hr).
L-HES/ND, L-HES mean compared to ND mean, resting (0 hr).
log2FC, log2 fold change.
pfp, percentage of false positives determined by RankProd.

Supplementary Table 6: Primers for quantitative RT-PCR

Gene	Fwd	Rev
ANK1	AAGTACCATGACTGAAGGGCTAGAG	CAGCCTCGGGCTGTTCTGT
B2M	TCTACTTTGAGTGCTGTCTCCATGT	AAGTTGCCAGCCCTCCTAGAG
CDCA7	TTCCGTGGAAGACATCCCCT	AGCTCTCCGTTCAGGGTTTC
CXCL13	TTGCCCCGTGGGAATG	GGGTCCACACACACAATTGACT
DNM3	CGGGATGAGATGCTTCGAAT	GGTGGCCGTGCTGATGTC
GATA3	TCTGGAGGAGGAATGCCAAT	CCGGGTTAAACGAGCTGTTC
GATA6	AGCACCAATCCCGAGAACAG	GGCGAGACTGACGCCTATGT
HDAC9	GGCATTAGAGGTACCCACAAATTG	TCTGAGGCAAAGGTGCAGACT
IFNG	TCCTGTGACTGTCTCACTTAATCCTT	TTAGGTTGGCTGCCTAGTTGG
IL17RB	CCTCTGGATAACAACAAAAGCAAG	AGTGTGGTGGTAGAAAAGGAAG
KCNK1	GGGATCACGTGTTACCTGCTACT	GCTCATGGAGTTCACAGAAGGTT
MAP3K8	GCCTAAGTGTTCAAATGACCG	GGCTGTAGATGTCTGCTTTGG
PLS3	TGGGAACCAAACCCTGACTTT	GATCTTCCAGGACATTGAGGGTAT
SGCE	CGGGAAGGCGTGGAAAA	GATTTCTGAATAGCACTGTGATGGA
SMAD7	AGGCTGTGTTGCTGTGAATCT	GCCAGATAATTCGTTCCCCCT
TNFSF11	TGGAGAGGAAATCAGCATCGA	AAAGCCCCAAAGTATGTTGCA
TOX	ATACTCAGGCCGCCATCAAG	CTTCGCCAAAGGTAGCGTTT
TWIST1	GCAAGATTCAGACCCTCAAGCT	TCGCTCTGGAGGACCTGGTA

Sequences are from 5′ to 3′.
