# Supplementary material for: Transcriptome analysis of Sézary syndrome and lymphocytic-variant hypereosinophilic syndrome T cells reveals common and divergent genes
Source: Oncotarget. 2019 Aug 20;10(49):5052–69. doi: 10.18632/oncotarget.27120 (PMC6707948; doi:10.18632/oncotarget.27120)
Supplement: Supplementary file 2 [file oncotarget-10-5052-s002.docx]

| **Supplementary Table 3: Genes and probes differentially expressed (0 hr) in L-HES vs. ND, but not in SS vs. ND** | | | | | | |
| --- | --- | --- | --- | --- | --- | --- |
|  |  |  |  |  |  |  |
|  |  |  | **SS/ND** | **SS/ND** | **L-HES/ND** | **L-HES/ND** |
| **Affymetrix ID** | **Entrez_IDs** | **Symbols** | **log2FC** | **pfp** | **log2FC** | **pfp** |
| 219557_s_at | 56675 | NRIP3 | -0.25 | 9.11E-01 | 5.71 | 1.29E-08 |
| 226517_at | 586 | BCAT1 | 0.62 | 4.77E-01 | 4.50 | 4.10E-06 |
| 225996_at | 164832 | LONRF2 | 0.59 | 4.85E-01 | 4.37 | 3.75E-06 |
| 222900_at | 56675 | NRIP3 | -0.32 | 7.35E-01 | 4.21 | 6.04E-06 |
| 224156_x_at | 55540 | IL17RB | 0.25 | 9.37E-01 | 4.19 | 5.89E-06 |
| 219255_x_at | 55540 | IL17RB | 0.25 | 9.66E-01 | 4.07 | 1.39E-05 |
| 225285_at | 586 | BCAT1 | 0.37 | 7.85E-01 | 4.03 | 1.26E-05 |
| 224361_s_at | 55540 | IL17RB | 0.34 | 8.12E-01 | 3.88 | 1.63E-05 |
| 212998_x_at | 3119 | HLA-DQB1 | 0.37 | 8.26E-01 | 3.67 | 2.27E-05 |
| 244413_at | 160365 | CLECL1 | 0.24 | 7.66E-01 | 3.66 | 3.24E-05 |
| 208059_at | 1237 | CCR8 | 0.72 | 2.60E-01 | 3.53 | 4.68E-05 |
| 213169_at | 9037 | SEMA5A | 0.01 | 1.06E+00 | 3.49 | 4.85E-05 |
| 206361_at | 11251 | PTGDR2 | 0.69 | 5.88E-01 | 3.48 | 6.21E-05 |
| 203029_s_at | 5799 | PTPRN2 | -0.04 | 1.20E+00 | 3.41 | 6.07E-05 |
| 235977_at | 164832 | LONRF2 | 0.29 | 7.85E-01 | 3.27 | 1.01E-04 |
| 228560_at | 55349 | CHDH | 0.27 | 8.92E-01 | 3.25 | 1.04E-04 |
| 225645_at | 26298 | EHF | 0.04 | 1.07E+00 | 3.19 | 3.56E-04 |
| 232968_at | 92565 | FANK1 | -0.88 | 1.27E-01 | 3.17 | 1.15E-04 |
| 238846_at | 8792 | TNFRSF11A | -0.53 | 3.84E-01 | 3.14 | 1.51E-04 |
| 219594_at | 4815 | NINJ2 | -1.04 | 1.41E-01 | 3.09 | 1.46E-04 |
| 203030_s_at | 5799 | PTPRN2 | 0.10 | 1.12E+00 | 3.02 | 1.86E-04 |
| 208791_at | 1191 | CLU | -1.23 | 6.43E-02 | 2.94 | 2.31E-04 |
| 209610_s_at | 6509 | SLC1A4 | 0.48 | 5.19E-01 | 2.93 | 3.62E-04 |
| 232028_at | 339500 | ZNF678 | 0.33 | 7.84E-01 | 2.86 | 3.11E-04 |
| 219806_s_at | 56935 | SMCO4 | -0.14 | 1.05E+00 | 2.84 | 3.29E-04 |
| 241879_at | 4026 | LPP | -0.74 | 2.13E-01 | 2.80 | 7.98E-04 |
| 208304_at | 1232 | CCR3 | -0.23 | 9.50E-01 | 2.77 | 4.51E-04 |
| 229954_at | 55349 | CHDH | 0.13 | 1.08E+00 | 2.75 | 4.32E-04 |
| 214452_at | 586 | BCAT1 | 0.30 | 8.09E-01 | 2.75 | 4.04E-04 |
| 208164_s_at | 3581 | IL9R | 0.69 | 2.75E-01 | 2.73 | 3.85E-04 |
| 213566_at | 6039 | RNASE6 | -0.14 | 4.98E-01 | 2.72 | 6.90E-04 |
| 208792_s_at | 1191 | CLU | -0.84 | 2.05E-01 | 2.68 | 4.38E-04 |
| 204014_at | 1846 | DUSP4 | -0.62 | 2.56E-01 | 2.66 | 8.99E-04 |
| 212810_s_at | 6509 | SLC1A4 | 0.32 | 7.40E-01 | 2.64 | 6.12E-04 |
| 220952_s_at | 54477 | PLEKHA5 | 1.18 | 6.33E-02 | 2.63 | 7.04E-04 |
| 209031_at | 23705 | CADM1 | 1.19 | 1.28E-01 | 2.59 | 6.22E-04 |
| 213385_at | 1124 | CHN2 | 0.69 | 3.29E-01 | 2.58 | 5.43E-04 |
| 212811_x_at | 6509 | SLC1A4 | 0.43 | 5.84E-01 | 2.57 | 6.55E-04 |
| 226034_at | 1846 | DUSP4 | -0.17 | 8.11E-01 | 2.55 | 8.16E-04 |
| 201909_at | 6192 | RPS4Y1 | 0.17 | 2.52E-01 | 2.50 | 2.95E-02 |
| 220051_at | 10942 | PRSS21 | 0.31 | 7.72E-01 | 2.50 | 7.38E-04 |
| 205900_at | 3848 | KRT1 | 0.96 | 1.85E-01 | 2.49 | 5.69E-03 |
| 213831_at | 3117 | HLA-DQA1 | -0.40 | 6.25E-01 | 2.43 | 7.42E-03 |
| 209016_s_at | 3855 | KRT7 | 0.95 | 2.52E-01 | 2.40 | 9.11E-04 |
| 205405_at | 9037 | SEMA5A | -0.13 | 1.17E+00 | 2.40 | 1.08E-03 |
| 229764_at | 285386 | TPRG1 | -0.87 | 1.74E-01 | 2.34 | 1.01E-03 |
| 1555465_at | 255231 | MCOLN2 | -0.26 | 9.19E-01 | 2.33 | 9.68E-04 |
| 218039_at | 51203 | NUSAP1 | 0.83 | 2.39E-01 | 2.33 | 1.21E-03 |
| 230252_at | 57121 | LPAR5 | 1.09 | 9.32E-02 | -2.35 | 2.56E-03 |
| 214551_s_at | 924 | CD7 | -0.72 | 8.45E-02 | -2.35 | 2.70E-03 |
| 236562_at | 90594 | ZNF439 | -0.32 | 8.69E-01 | -2.36 | 2.48E-03 |
| 203543_s_at | 687 | KLF9 | -0.20 | 9.78E-01 | -2.36 | 2.61E-03 |
| 202446_s_at | 5359 | PLSCR1 | 0.22 | 9.99E-01 | -2.39 | 2.29E-03 |
| 221602_s_at | 9214 | FCMR | -0.30 | 7.52E-01 | -2.39 | 2.47E-03 |
| 1555579_s_at | 5797 | PTPRM | 0.02 | 1.01E+00 | -2.42 | 2.40E-03 |
| 219457_s_at | 79890 | RIN3 | 0.40 | 6.48E-01 | -2.44 | 2.47E-03 |
| 230378_at | 92304 | SCGB3A1 | -0.31 | 6.13E-01 | -2.45 | 2.11E-03 |
| 201656_at | 3655 | ITGA6 | -0.92 | 1.90E-01 | -2.47 | 2.10E-03 |
| 217232_x_at | 3043 | HBB | -0.11 | 1.19E+00 | -2.47 | 4.32E-03 |
| 241859_at | 5334 | PLCL1 | -0.16 | 7.00E-01 | -2.48 | 2.12E-03 |
| 202933_s_at | 7525 | YES1 | -0.53 | 3.88E-01 | -2.48 | 2.00E-03 |
| 219132_at | 57161 | PELI2 | 0.83 | 2.93E-01 | -2.49 | 1.90E-03 |
| 203939_at | 4907 | NT5E | -0.31 | 4.77E-01 | -2.49 | 2.08E-03 |
| 238649_at | 26207 | PITPNC1 | -0.66 | 2.84E-01 | -2.49 | 2.01E-03 |
| 202371_at | 79921 | TCEAL4 | -0.18 | 8.92E-01 | -2.49 | 2.48E-03 |
| 220560_at | 29125 | C11orf21 | -0.83 | 2.11E-01 | -2.50 | 1.91E-03 |
| 200602_at | 351 | APP | -0.60 | 3.73E-01 | -2.51 | 1.86E-03 |
| 205929_at | 10223 | GPA33 | 0.78 | 2.83E-01 | -2.52 | 1.72E-03 |
| 201722_s_at | 2589 | GALNT1 | -0.54 | 3.05E-01 | -2.52 | 2.00E-03 |
| 236301_at | 22806 | IKZF3 | -0.78 | 2.70E-01 | -2.56 | 2.11E-03 |
| 1568618_a_at | 2589 | GALNT1 | -0.54 | 3.23E-01 | -2.56 | 1.95E-03 |
| 229070_at | 84830 | ADTRP | -0.24 | 4.40E-01 | -2.58 | 1.72E-03 |
| 201310_s_at | 9315 | NREP | 1.10 | 1.46E-01 | -2.59 | 1.12E-03 |
| 37145_at | 10578 | GNLY | -0.86 | 1.98E-01 | -2.59 | 2.11E-03 |
| 202430_s_at | 5359 | PLSCR1 | 0.41 | 6.43E-01 | -2.61 | 1.56E-03 |
| 223380_s_at | 26524 | LATS2 | 0.07 | 1.05E+00 | -2.61 | 1.73E-03 |
| 1564150_a_at | 256021 | LINC01619 | 0.49 | 3.28E-01 | -2.62 | 1.30E-03 |
| 226388_at | 6920 | TCEA3 | 1.04 | 1.04E-01 | -2.63 | 1.25E-03 |
| 218510_x_at | 54463 | RETREG1 | 0.29 | 7.40E-01 | -2.64 | 1.31E-03 |
| 227533_at | 55103 | RALGPS2 | -0.42 | 4.68E-01 | -2.67 | 1.29E-03 |
| 229725_at | 23305 | ACSL6 | 0.10 | 8.87E-01 | -2.75 | 1.10E-03 |
| 210915_x_at | 28639 | TRBC1 | 0.05 | 1.05E+00 | -2.76 | 4.46E-03 |
| 214039_s_at | 55353 | LAPTM4B | -0.96 | 1.54E-01 | -2.77 | 1.16E-03 |
| 224140_at | 246734 | NPCDR1 | 0.28 | 8.51E-01 | -2.82 | 1.11E-03 |
| 222101_s_at | 8642 | DCHS1 | 0.17 | 6.13E-01 | -2.84 | 7.89E-04 |
| 226433_at | 114804 | RNF157 | -1.13 | 1.25E-01 | -2.87 | 8.30E-04 |
| 201242_s_at | 481 | ATP1B1 | -0.56 | 1.54E-01 | -2.87 | 1.02E-03 |
| 60471_at | 79890 | RIN3 | 0.32 | 8.39E-01 | -2.91 | 7.08E-04 |
| 213193_x_at | 28639 | TRBC1 | -0.01 | 1.11E+00 | -2.94 | 2.90E-03 |
| 221648_s_at | 79814 | AGMAT | 0.66 | 2.28E-01 | -2.99 | 6.66E-04 |
| 1564435_a_at | 140807 | KRT72 | -0.16 | 1.14E+00 | -3.00 | 3.79E-04 |
| 203627_at | 3480 | IGF1R | -0.30 | 2.56E-01 | -3.01 | 4.75E-04 |
| 238577_s_at | 128553 | TSHZ2 | 0.28 | 8.59E-01 | -3.07 | 8.30E-04 |
| 202336_s_at | 5066 | PAM | -0.55 | 4.39E-01 | -3.07 | 5.27E-04 |
| 229390_at | 441168 | CALHM6 | -1.08 | 1.03E-01 | -3.08 | 3.98E-04 |
| 218312_s_at | 65982 | ZSCAN18 | -0.48 | 5.03E-01 | -3.13 | 5.23E-04 |
| 1557733_a_at | 100506915 | CHRM3-AS2 | -1.06 | 6.63E-02 | -3.14 | 3.91E-04 |
| 209116_x_at | 3043 | HBB | -0.11 | 1.19E+00 | -3.18 | 2.39E-04 |
| 214255_at | 57194 | ATP10A | -0.36 | 7.00E-01 | -3.20 | 3.66E-04 |
| 201417_at | 6659 | SOX4 | 1.28 | 7.44E-02 | -3.21 | 4.67E-04 |
| 218532_s_at | 54463 | RETREG1 | 0.10 | 1.05E+00 | -3.22 | 3.63E-04 |
| 243940_at | 128553 | TSHZ2 | 0.85 | 2.33E-01 | -3.31 | 2.42E-04 |
| 228297_at | 1266 | CNN3 | 0.20 | 8.07E-01 | -3.31 | 3.07E-04 |
| 235616_at | 128553 | TSHZ2 | 0.47 | 6.43E-01 | -3.33 | 3.90E-04 |
| 211696_x_at | 3043 | HBB | -0.22 | 9.80E-01 | -3.43 | 1.77E-04 |
| 225081_s_at | 55536 | CDCA7L | 0.98 | 1.55E-01 | -3.46 | 1.71E-04 |
| 205934_at | 5334 | PLCL1 | -0.06 | 8.96E-01 | -3.54 | 1.74E-04 |
| 208637_x_at | 87 | ACTN1 | -0.55 | 2.84E-01 | -3.61 | 1.67E-04 |
| 227210_at | 57713 | SFMBT2 | 0.60 | 3.76E-01 | -3.62 | 1.45E-04 |
| 220112_at | 79722 | ANKRD55 | -0.96 | 9.85E-02 | -3.74 | 1.17E-04 |
| 206337_at | 1236 | CCR7 | 0.59 | 3.29E-01 | -3.87 | 7.81E-05 |
| 203628_at | 3480 | IGF1R | -0.24 | 3.09E-01 | -4.07 | 5.26E-05 |
| 206492_at | 2272 | FHIT | 0.60 | 3.24E-01 | -4.08 | 5.03E-05 |
| 203413_at | 4753 | NELL2 | 0.50 | 4.91E-01 | -4.10 | 5.09E-05 |
| 225330_at | 3480 | IGF1R | -0.93 | 1.25E-01 | -4.17 | 4.64E-05 |
| 213005_s_at | 23189 | KANK1 | -0.93 | 1.60E-01 | -4.31 | 4.46E-05 |
| 206828_at | 7294 | TXK | -0.51 | 2.27E-01 | -4.52 | 2.08E-05 |
| 202599_s_at | 8204 | NRIP1 | -0.35 | 6.49E-01 | -4.72 | 1.10E-05 |
| 206150_at | 939 | CD27 | 1.02 | 8.46E-02 | -4.93 | 5.11E-06 |
| 231798_at | 9241 | NOG | -0.72 | 2.00E-01 | -4.99 | 5.03E-06 |
| 202600_s_at | 8204 | NRIP1 | -0.64 | 3.02E-01 | -5.01 | 4.52E-06 |
| 208636_at | 87 | ACTN1 | -0.75 | 1.43E-01 | -5.39 | 6.49E-07 |
| 209841_s_at | 54674 | LRRN3 | -0.98 | 1.47E-01 | -5.97 | 1.11E-07 |
| 209840_s_at | 54674 | LRRN3 | -1.04 | 1.06E-01 | -6.19 | 9.64E-08 |

This table corresponds to Figure 4C, and includes probes with differential expression of log2FC ≥ |2.33| (or 5 fold) and percentage of false prediction (pfp) < 0.05 in L-HES, as determined by the RankProduct method.
