# Supplementary material for: Transcriptome analysis of Sézary syndrome and lymphocytic-variant hypereosinophilic syndrome T cells reveals common and divergent genes
Source: Oncotarget. 2019 Aug 20;10(49):5052–69. doi: 10.18632/oncotarget.27120 (PMC6707948; doi:10.18632/oncotarget.27120)
Supplement: Supplementary file 3 [file oncotarget-10-5052-s003.docx]

| **Supplementary Table 4: Genes and probes differentially expressed in both SS and LHES (0 hr)** | | | | | | | |
| --- | --- | --- | --- | --- | --- | --- | --- |
|  |  |  |  |  |  |  |  |
|  | **Affymetrix ID** | **Entrez_ID** | **Gene Symbol** | **SS log2FC** | **SS pfp** | **LHES log2FC** | **LHES pfp** |
| **Group 1** | 225436_at | 58489 | *ABHD17C* | 1.42 | 3.16E-02 | 1.42 | 2.02E-02 |
| SS up/LHES up | 207111_at | 2015 | *ADGRE1* | 1.57 | 1.75E-02 | 1.97 | 1.69E-02 |
|  | 208376_at | 1233 | *CCR4* | 1.37 | 3.91E-02 | 1.67 | 8.76E-03 |
|  | 224428_s_at | 83879 | *CDCA7* | 2.09 | 5.73E-03 | 2.55 | 9.54E-04 |
|  | 201131_s_at | 999 | *CDH1* | 4.73 | 6.45E-06 | 4.66 | 1.79E-06 |
|  | 212942_s_at | 57214 | *CEMIP* | 3.45 | 2.55E-04 | 2.29 | 1.45E-03 |
|  | 225129_at | 221184 | *CPNE2* | 2.08 | 2.53E-02 | 2.55 | 6.26E-04 |
|  | 208303_s_at | 64109 | *CRLF2* | 1.62 | 3.92E-02 | 1.22 | 3.81E-02 |
|  | 238021_s_at | 643911 | *CRNDE* | 1.83 | 1.76E-02 | 2.14 | 4.34E-03 |
|  | 203881_s_at | 1756 | *DMD* | 1.64 | 2.60E-02 | 2.41 | 1.32E-03 |
|  | 1558501_at | 26052 | *DNM3* | 4.73 | 7.14E-06 | 5.02 | 8.90E-07 |
|  | 1558502_s_at | 26052 | *DNM3* | 2.56 | 1.69E-03 | 2.57 | 6.60E-04 |
|  | 209839_at | 26052 | *DNM3* | 5.37 | 6.30E-07 | 4.84 | 1.86E-06 |
|  | 235745_at | 2081 | *ERN1* | 1.78 | 3.45E-02 | 1.48 | 1.51E-02 |
|  | 209603_at | 2625 | *GATA3* | 1.49 | 2.27E-02 | 1.87 | 3.98E-03 |
|  | 1557167_at | 493812 | *HCG11* | 1.49 | 2.09E-02 | 1.75 | 2.03E-02 |
|  | 1557169_x_at | 493812 | *HCG11* | 1.57 | 1.66E-02 | 1.51 | 3.84E-02 |
|  | 1560573_at | 387895 | *LINC00944* | 1.96 | 8.28E-03 | 1.86 | 5.05E-03 |
|  | 204784_s_at | 4291 | *MLF1* | 2.86 | 9.68E-04 | 2.21 | 2.56E-03 |
|  | 224799_at | 54602 | *NDFIP2* | 1.45 | 2.40E-02 | 1.28 | 2.06E-02 |
|  | 224802_at | 54602 | *NDFIP2* | 1.95 | 6.51E-03 | 1.81 | 5.66E-03 |
|  | 212094_at | 23089 | *PEG10* | 4.06 | 6.87E-05 | 1.49 | 3.25E-02 |
|  | 204688_at | 8910 | *SGCE* | 3.66 | 1.40E-04 | 1.67 | 2.48E-02 |
|  | 219496_at | 65124 | *SOWAHC* | 1.51 | 2.53E-02 | 1.86 | 3.78E-03 |
|  | 227034_at | 65124 | *SOWAHC* | 1.79 | 1.23E-02 | 1.67 | 8.07E-03 |
|  | 209198_s_at | 23208 | *SYT11* | 1.31 | 4.11E-02 | 1.80 | 4.31E-03 |
|  | 210643_at | 8600 | *TNFSF11* | 3.24 | 4.53E-04 | 2.89 | 3.21E-04 |
|  | 211153_s_at | 8600 | *TNFSF11* | 2.24 | 3.44E-03 | 1.36 | 1.96E-02 |
|  | 241808_at | 51101 | *ZC2HC1A* | 1.54 | 2.97E-02 | 1.32 | 3.54E-02 |
|  | 228280_at | 92092 | *ZC3HAV1L* | 2.07 | 5.96E-03 | 2.61 | 8.45E-04 |
|  | 206448_at | 22891 | *ZNF365* | 2.15 | 4.09E-03 | 4.55 | 2.13E-06 |
|  | 1558105_a_at | NA | *NA* | 1.24 | 4.47E-02 | 1.79 | 4.37E-03 |
|  | 226550_at | NA | *NA* | 1.28 | 4.15E-02 | 1.68 | 7.40E-03 |
|  | 232504_at | NA | *NA* | 2.04 | 5.51E-03 | 1.13 | 4.53E-02 |
|  | 236215_at | NA | *NA* | 1.35 | 3.39E-02 | 1.77 | 6.08E-03 |
| **Group 2** | 209160_at | 8644 | *AKR1C3* | 1.94 | 7.15E-03 | -2.42 | 4.18E-03 |
| SS up/LHES down | 205390_s_at | 286 | *ANK1* | 2.65 | 1.42E-03 | -1.56 | 2.49E-02 |
|  | 208353_x_at | 286 | *ANK1* | 1.81 | 9.17E-03 | -1.41 | 3.02E-02 |
|  | 235463_s_at | 253782 | *CERS6* | 1.53 | 2.60E-02 | -1.60 | 1.58E-02 |
|  | 212624_s_at | 1123 | *CHN1* | 2.25 | 5.02E-03 | -2.05 | 4.48E-03 |
|  | 205898_at | 1524 | *CX3CR1* | 2.65 | 1.90E-03 | -1.72 | 1.29E-02 |
|  | 231093_at | 115352 | *FCRL3* | 3.93 | 7.81E-05 | -2.15 | 5.87E-03 |
|  | 208524_at | 2838 | *GPR15* | 2.74 | 1.29E-03 | -2.10 | 4.61E-03 |
|  | 231929_at | 22807 | *IKZF2* | 3.10 | 5.90E-04 | -1.45 | 4.13E-02 |
|  | 243362_s_at | 641518 | *LEF1-AS1* | 1.72 | 2.56E-02 | -3.84 | 9.00E-05 |
|  | 243363_at | 641518 | *LEF1-AS1* | 1.87 | 1.66E-02 | -1.92 | 7.27E-03 |
|  | 204798_at | 4602 | *MYB* | 1.88 | 7.34E-03 | -1.51 | 2.31E-02 |
|  | 228461_at | 344558 | *SH3RF3* | 1.53 | 1.93E-02 | -1.71 | 1.17E-02 |
|  | 202260_s_at | 6812 | *STXBP1* | 1.23 | 4.16E-02 | -1.62 | 1.91E-02 |
|  | 244741_s_at | 100128252 | *ZNF667-AS1* | 3.07 | 5.54E-04 | -1.35 | 4.04E-02 |
|  | 209813_x_at | NA | *NA* | 2.29 | 3.06E-03 | -2.56 | 3.96E-03 |
|  | 211144_x_at | NA | *NA* | 2.25 | 3.27E-03 | -2.11 | 8.04E-03 |
|  | 214414_x_at | NA | *NA* | 2.09 | 2.56E-02 | -3.73 | 4.85E-05 |
|  | 215806_x_at | NA | *NA* | 2.25 | 3.31E-03 | -2.58 | 2.67E-03 |
|  | 216920_s_at | NA | *NA* | 2.04 | 5.71E-03 | -2.84 | 2.06E-03 |
|  | 217414_x_at | NA | *NA* | 1.76 | 4.78E-02 | -2.48 | 3.81E-03 |
|  | 242551_at | NA | *NA* | 1.39 | 3.64E-02 | -1.61 | 1.91E-02 |
| **Group 3** | 203196_at | 10257 | *ABCC4* | -1.65 | 1.01E-02 | 1.38 | 2.02E-02 |
| SS down/LHES up | 224996_at | 444 | *ASPH* | -1.43 | 2.53E-02 | 1.24 | 3.52E-02 |
|  | 204780_s_at | 355 | *FAS* | -1.22 | 4.98E-02 | 1.22 | 3.46E-02 |
|  | 215719_x_at | 355 | *FAS* | -1.50 | 2.74E-02 | 1.31 | 3.06E-02 |
|  | 216252_x_at | 355 | *FAS* | -1.31 | 4.39E-02 | 1.38 | 1.73E-02 |
|  | 201137_s_at | 3115 | *HLA-DPB1* | -1.55 | 2.34E-02 | 2.33 | 8.42E-04 |
|  | 232397_at | 101927482 | *LOC101927482* | -2.68 | 6.10E-04 | 1.98 | 4.42E-03 |
|  | 232752_at | 100287616 | *LOXL1-AS1* | -1.85 | 5.92E-03 | 1.89 | 1.59E-02 |
|  | 228708_at | 5874 | *RAB27B* | -1.77 | 7.13E-03 | 2.67 | 5.78E-04 |
|  | 204759_at | 1102 | *RCBTB2* | -1.34 | 3.89E-02 | 2.04 | 2.35E-03 |
|  | 202988_s_at | 5996 | *RGS1* | -2.02 | 3.46E-03 | 2.33 | 1.32E-03 |
|  | 216834_at | 5996 | *RGS1* | -2.45 | 1.04E-03 | 1.83 | 4.45E-03 |
|  | 212671_s_at | NA | *NA* | -1.27 | 4.43E-02 | 3.89 | 1.50E-05 |
|  | 230292_at | NA | *NA* | -1.56 | 1.44E-02 | 1.65 | 7.61E-03 |
|  | 243931_at | NA | *NA* | -1.22 | 4.44E-02 | 1.09 | 4.27E-02 |
| **Group 4** | 209993_at | 5243 | *ABCB1* | -1.74 | 2.64E-02 | -3.02 | 5.18E-04 |
| SS down/LHES down | 213808_at | 8745 | *ADAM23* | -2.09 | 3.24E-03 | -1.91 | 8.32E-03 |
|  | 244463_at | 8745 | *ADAM23* | -1.34 | 3.02E-02 | -1.67 | 8.26E-03 |
|  | 206170_at | 154 | *ADRB2* | -1.73 | 8.02E-03 | -2.17 | 4.50E-03 |
|  | 204446_s_at | 240 | *ALOX5* | -2.78 | 4.81E-04 | -2.34 | 2.67E-03 |
|  | 209870_s_at | 321 | *APBA2* | -1.23 | 4.10E-02 | -1.81 | 9.81E-03 |
|  | 209871_s_at | 321 | *APBA2* | -2.29 | 1.61E-03 | -3.24 | 3.14E-04 |
|  | 212599_at | 26053 | *AUTS2* | -4.95 | 2.58E-07 | -3.40 | 1.12E-03 |
|  | 210121_at | 8707 | *B3GALT2* | -3.27 | 1.20E-04 | -3.48 | 2.28E-04 |
|  | 217452_s_at | 8707 | *B3GALT2* | -3.75 | 2.38E-05 | -4.15 | 5.86E-05 |
|  | 221234_s_at | 60468 | *BACH2* | -1.37 | 2.80E-02 | -2.39 | 2.57E-03 |
|  | 209406_at | 9532 | *BAG2* | -1.24 | 3.80E-02 | -1.86 | 9.58E-03 |
|  | 225606_at | 10018 | *BCL2L11* | -1.18 | 4.93E-02 | -1.29 | 4.97E-02 |
|  | 201849_at | 664 | *BNIP3* | -1.21 | 4.77E-02 | -2.01 | 6.84E-03 |
|  | 228570_at | 121551 | *BTBD11* | -2.29 | 1.81E-03 | -3.50 | 2.13E-04 |
|  | 238692_at | 121551 | *BTBD11* | -2.59 | 7.20E-04 | -2.13 | 3.70E-03 |
|  | 242520_s_at | 339541 | *C1orf228* | -1.89 | 5.92E-03 | -1.96 | 2.43E-02 |
|  | 218309_at | 55450 | *CAMK2N1* | -2.20 | 2.13E-03 | -1.78 | 1.26E-02 |
|  | 205476_at | 6364 | *CCL20* | -2.26 | 1.79E-03 | -1.18 | 2.99E-02 |
|  | 1405_i_at | 6352 | *CCL5* | -4.76 | 3.81E-07 | -4.52 | 1.59E-05 |
|  | 1555759_a_at | 6352 | *CCL5* | -4.18 | 4.43E-06 | -4.12 | 5.02E-05 |
|  | 204655_at | 6352 | *CCL5* | -4.21 | 4.50E-06 | -4.36 | 3.14E-05 |
|  | 206983_at | 1235 | *CCR6* | -2.47 | 3.32E-03 | -2.86 | 9.98E-04 |
|  | 204995_at | 8851 | *CDK5R1* | -1.63 | 2.50E-02 | -1.29 | 4.55E-02 |
|  | 203973_s_at | 1052 | *CEBPD* | -2.10 | 2.83E-03 | -2.64 | 1.51E-03 |
|  | 1556209_at | 9976 | *CLEC2B* | -1.83 | 7.76E-03 | -2.20 | 1.06E-02 |
|  | 206244_at | 1378 | *CR1* | -1.36 | 2.72E-02 | -1.96 | 5.17E-03 |
|  | 208488_s_at | 1378 | *CR1* | -1.44 | 2.00E-02 | -1.96 | 9.80E-03 |
|  | 217552_x_at | 1378 | *CR1* | -2.75 | 4.75E-04 | -3.23 | 5.51E-04 |
|  | 244313_at | 1378 | *CR1* | -2.83 | 4.50E-04 | -3.65 | 1.19E-04 |
|  | 205544_s_at | 1380 | *CR2* | -1.38 | 2.47E-02 | -2.16 | 2.80E-03 |
|  | 206974_at | 10663 | *CXCR6* | -2.36 | 1.41E-03 | -1.69 | 1.24E-02 |
|  | 211469_s_at | 10663 | *CXCR6* | -2.56 | 7.88E-04 | -1.35 | 4.93E-02 |
|  | 230866_at | 10800 | *CYSLTR1* | -2.29 | 1.81E-03 | -3.27 | 3.67E-04 |
|  | 231747_at | 10800 | *CYSLTR1* | -2.11 | 9.98E-03 | -3.70 | 1.18E-04 |
|  | 219452_at | 64174 | *DPEP2* | -2.31 | 1.81E-03 | -2.45 | 2.40E-03 |
|  | 231776_at | 8320 | *EOMES* | -2.23 | 1.96E-03 | -1.66 | 1.45E-02 |
|  | 232164_s_at | 83481 | *EPPK1* | -1.48 | 2.73E-02 | -2.97 | 7.75E-04 |
|  | 232165_at | 83481 | *EPPK1* | -1.29 | 4.62E-02 | -2.52 | 2.11E-03 |
|  | 203989_x_at | 2149 | *F2R* | -2.34 | 1.56E-03 | -1.42 | 3.26E-02 |
|  | 230923_at | 407738 | *FAM19A1* | -1.68 | 9.42E-03 | -1.83 | 9.60E-03 |
|  | 206404_at | 2254 | *FGF9* | -2.38 | 1.29E-03 | -1.77 | 1.50E-02 |
|  | 223836_at | 83888 | *FGFBP2* | -1.44 | 3.41E-02 | -2.33 | 6.51E-03 |
|  | 201540_at | 2273 | *FHL1* | -2.16 | 2.51E-03 | -3.24 | 3.53E-04 |
|  | 210298_x_at | 2273 | *FHL1* | -1.44 | 2.35E-02 | -1.92 | 8.11E-03 |
|  | 210299_s_at | 2273 | *FHL1* | -2.06 | 3.08E-03 | -2.34 | 2.57E-03 |
|  | 214505_s_at | 2273 | *FHL1* | -1.58 | 1.48E-02 | -1.84 | 9.59E-03 |
|  | 238581_at | 115362 | *GBP5* | -2.10 | 8.26E-03 | -3.51 | 2.14E-04 |
|  | 223423_at | 26996 | *GPR160* | -1.91 | 5.95E-03 | -1.96 | 8.55E-03 |
|  | 213142_x_at | 54103 | *GSAP* | -2.91 | 3.94E-04 | -1.89 | 2.08E-02 |
|  | 222150_s_at | 54103 | *GSAP* | -3.04 | 2.54E-04 | -2.03 | 1.81E-02 |
|  | 202554_s_at | 2947 | *GSTM3* | -1.53 | 3.45E-02 | -2.32 | 4.20E-03 |
|  | 205488_at | 3001 | *GZMA* | -3.61 | 4.17E-05 | -2.42 | 2.65E-03 |
|  | 210321_at | 2999 | *GZMH* | -1.24 | 4.97E-02 | -1.97 | 5.16E-03 |
|  | 206666_at | 3003 | *GZMK* | -4.77 | 4.49E-07 | -3.92 | 7.48E-05 |
|  | 225792_at | 51361 | *HOOK1* | -1.71 | 1.49E-02 | -2.90 | 8.15E-04 |
|  | 211597_s_at | 84525 | *HOPX* | -3.46 | 8.21E-05 | -1.83 | 1.34E-02 |
|  | 205453_at | 3212 | *HOXB2* | -1.66 | 1.36E-02 | -1.62 | 3.52E-02 |
|  | 214453_s_at | 10561 | *IFI44* | -2.62 | 6.32E-04 | -1.56 | 2.05E-02 |
|  | 204439_at | 10964 | *IFI44L* | -1.94 | 5.79E-03 | -1.54 | 1.48E-02 |
|  | 210095_s_at | 3486 | *IGFBP3* | -2.15 | 2.26E-03 | -1.55 | 4.33E-02 |
|  | 206618_at | 8809 | *IL18R1* | -1.85 | 6.57E-03 | -2.14 | 3.15E-03 |
|  | 207072_at | 8807 | *IL18RAP* | -2.00 | 3.38E-03 | -2.58 | 2.05E-03 |
|  | 216857_at | 51561 | *IL23A* | -1.77 | 7.09E-03 | -2.16 | 4.34E-03 |
|  | 234377_at | 51561 | *IL23A* | -2.65 | 6.31E-04 | -3.17 | 3.92E-04 |
|  | 205884_at | 3676 | *ITGA4* | -2.68 | 6.27E-04 | -2.81 | 2.87E-03 |
|  | 205885_s_at | 3676 | *ITGA4* | -2.81 | 4.78E-04 | -3.26 | 1.23E-03 |
|  | 212813_at | 83700 | *JAM3* | -1.36 | 2.48E-02 | -1.80 | 1.05E-02 |
|  | 228094_at | 120425 | *JAML* | -1.97 | 4.03E-03 | -2.53 | 1.95E-03 |
|  | 205051_s_at | 3815 | *KIT* | -2.17 | 2.40E-03 | -2.47 | 2.11E-03 |
|  | 214470_at | 3820 | *KLRB1* | -4.65 | 4.45E-07 | -4.29 | 3.38E-05 |
|  | 208949_s_at | 3958 | *LGALS3* | -2.30 | 6.14E-03 | -3.96 | 8.80E-05 |
|  | 1569872_a_at | 64788 | *LMF1* | -1.49 | 2.53E-02 | -2.87 | 9.13E-04 |
|  | 227984_at | 64788 | *LMF1* | -1.38 | 3.12E-02 | -2.90 | 7.56E-04 |
|  | 240890_at | 643733 | *LOC643733* | -1.27 | 3.71E-02 | -1.35 | 4.07E-02 |
|  | 223414_s_at | 55646 | *LYAR* | -1.28 | 3.25E-02 | -2.18 | 4.19E-03 |
|  | 213975_s_at | 4069 | *LYZ* | -2.42 | 4.16E-03 | -2.01 | 5.47E-03 |
|  | 228423_at | 79884 | *MAP9* | -1.93 | 4.92E-03 | -1.89 | 1.57E-02 |
|  | 1569136_at | 11320 | *MGAT4A* | -1.30 | 3.00E-02 | -2.11 | 5.41E-03 |
|  | 213915_at | 4818 | *NKG7* | -1.88 | 6.36E-03 | -1.81 | 1.29E-02 |
|  | 209569_x_at | 27065 | *NSG1* | -2.48 | 9.58E-04 | -3.22 | 3.11E-04 |
|  | 209570_s_at | 27065 | *NSG1* | -1.93 | 4.89E-03 | -3.01 | 5.92E-04 |
|  | 213533_at | 27065 | *NSG1* | -1.70 | 9.22E-03 | -1.67 | 1.47E-02 |
|  | 228298_at | 91523 | *PCED1B* | -2.51 | 9.12E-04 | -1.43 | 2.90E-02 |
|  | 213652_at | 5125 | *PCSK5* | -1.33 | 2.84E-02 | -2.36 | 2.56E-03 |
|  | 1557166_at | 27250 | *PDCD4* | -1.54 | 1.49E-02 | -1.78 | 8.08E-03 |
|  | 225688_s_at | 90102 | *PHLDB2* | -1.68 | 1.67E-02 | -2.27 | 3.76E-03 |
|  | 219014_at | 51316 | *PLAC8* | -3.00 | 2.87E-04 | -3.43 | 2.18E-04 |
|  | 213222_at | 23236 | *PLCB1* | -2.17 | 2.26E-03 | -1.96 | 6.56E-03 |
|  | 219024_at | 59338 | *PLEKHA1* | -1.29 | 3.50E-02 | -1.43 | 2.63E-02 |
|  | 226247_at | 59338 | *PLEKHA1* | -1.73 | 8.13E-03 | -1.46 | 2.69E-02 |
|  | 212235_at | 23129 | *PLXND1* | -1.39 | 2.47E-02 | -1.75 | 1.31E-02 |
|  | 38671_at | 23129 | *PLXND1* | -1.73 | 7.80E-03 | -1.70 | 1.12E-02 |
|  | 222449_at | 56937 | *PMEPA1* | -2.21 | 2.03E-03 | -1.38 | 2.49E-02 |
|  | 229809_at | 5463 | *POU6F1* | -1.74 | 7.76E-03 | -1.79 | 8.55E-03 |
|  | 235085_at | 157285 | *PRAG1* | -2.83 | 4.54E-04 | -2.81 | 9.40E-04 |
|  | 214617_at | 5551 | *PRF1* | -2.46 | 1.04E-03 | -1.81 | 1.21E-02 |
|  | 209815_at | 5727 | *PTCH1* | -2.91 | 3.95E-04 | -2.84 | 1.12E-03 |
|  | 208820_at | 5747 | *PTK2* | -1.39 | 2.40E-02 | -1.33 | 4.62E-02 |
|  | 204201_s_at | 5783 | *PTPN13* | -1.92 | 5.94E-03 | -2.28 | 2.01E-03 |
|  | 230230_at | 5775 | *PTPN4* | -1.35 | 2.53E-02 | -1.63 | 4.78E-02 |
|  | 228109_at | 5924 | *RASGRF2* | -1.97 | 5.21E-03 | -2.86 | 8.13E-04 |
|  | 235816_s_at | 266747 | *RGL4* | -1.20 | 4.63E-02 | -1.31 | 3.91E-02 |
|  | 209545_s_at | 8767 | *RIPK2* | -1.42 | 2.07E-02 | -2.33 | 2.60E-03 |
|  | 217865_at | 55819 | *RNF130* | -1.98 | 4.56E-03 | -1.60 | 2.48E-02 |
|  | 210426_x_at | 6095 | *RORA* | -1.53 | 1.60E-02 | -1.90 | 9.53E-03 |
|  | 210479_s_at | 6095 | *RORA* | -1.49 | 1.72E-02 | -2.01 | 8.10E-03 |
|  | 226682_at | 6095 | *RORA* | -1.25 | 3.83E-02 | -1.56 | 2.02E-02 |
|  | 228806_at | 6097 | *RORC* | -1.43 | 2.08E-02 | -1.49 | 2.47E-02 |
|  | 221523_s_at | 58528 | *RRAGD* | -1.62 | 1.62E-02 | -1.85 | 8.28E-03 |
|  | 241365_at | 6304 | *SATB1* | -2.74 | 5.95E-04 | -1.23 | 5.07E-02 |
|  | 203408_s_at | 6304 | *SATB1* | -2.23 | 2.12E-03 | -0.83 | 2.08E-01 |
|  | 244267_at | 6304 | *SATB1* | -0.75 | 1.86E-01 | -1.35 | 4.03E-02 |
|  | 226169_at | 81846 | *SBF2* | -2.12 | 2.43E-03 | -1.50 | 2.22E-02 |
|  | 240830_at | 677769 | *SCARNA17* | -1.25 | 3.80E-02 | -1.42 | 3.63E-02 |
|  | 218793_s_at | 6322 | *SCML1* | -1.82 | 9.26E-03 | -3.32 | 3.06E-04 |
|  | 201462_at | 9805 | *SCRN1* | -1.16 | 4.99E-02 | -1.76 | 9.60E-03 |
|  | 211474_s_at | 5269 | *SERPINB6* | -1.39 | 2.47E-02 | -1.51 | 2.24E-02 |
|  | 224928_at | 80854 | *SETD7* | -1.34 | 2.95E-02 | -2.92 | 6.81E-04 |
|  | 236220_at | 117247 | *SLC16A10* | -1.60 | 1.21E-02 | -2.65 | 1.51E-03 |
|  | 223044_at | 30061 | *SLC40A1* | -1.67 | 1.54E-02 | -2.05 | 1.58E-02 |
|  | 204790_at | 4092 | *SMAD7* | -3.11 | 2.56E-04 | -2.84 | 9.34E-04 |
|  | 223276_at | 85027 | *SMIM3* | -1.66 | 1.75E-02 | -1.40 | 3.25E-02 |
|  | 223027_at | 51429 | *SNX9* | -1.71 | 1.98E-02 | -2.90 | 7.83E-04 |
|  | 223028_s_at | 51429 | *SNX9* | -1.79 | 1.65E-02 | -2.97 | 6.57E-04 |
|  | 209436_at | 10418 | *SPON1* | -1.41 | 4.98E-02 | -2.21 | 3.95E-03 |
|  | 213993_at | 10418 | *SPON1* | -1.69 | 2.48E-02 | -2.60 | 1.28E-03 |
|  | 213994_s_at | 10418 | *SPON1* | -1.92 | 1.36E-02 | -2.72 | 1.06E-03 |
|  | 213400_s_at | 6907 | *TBL1X* | -2.05 | 7.72E-03 | -1.50 | 2.40E-02 |
|  | 217975_at | 51186 | *TCEAL9* | -1.55 | 1.38E-02 | -1.28 | 3.42E-02 |
|  | 236561_at | 7046 | *TGFBR1* | -1.18 | 4.97E-02 | -1.33 | 3.73E-02 |
|  | 207334_s_at | 7048 | *TGFBR2* | -1.20 | 4.62E-02 | -2.24 | 4.22E-03 |
|  | 208944_at | 7048 | *TGFBR2* | -2.06 | 3.30E-03 | -2.53 | 1.89E-03 |
|  | 226226_at | 120224 | *TMEM45B* | -1.30 | 3.98E-02 | -2.04 | 6.79E-03 |
|  | 230323_s_at | 120224 | *TMEM45B* | -2.12 | 2.56E-03 | -3.46 | 2.25E-04 |
|  | 238429_at | 137835 | *TMEM71* | -1.75 | 7.91E-03 | -1.47 | 2.68E-02 |
|  | 223501_at | 10673 | *TNFSF13B* | -1.48 | 2.49E-02 | -3.30 | 3.15E-04 |
|  | 223502_s_at | 10673 | *TNFSF13B* | -1.55 | 2.07E-02 | -3.10 | 5.29E-04 |
|  | 215797_at | 28683 | *TRAV8-3* | -2.47 | 9.67E-04 | -2.08 | 8.94E-03 |
|  | 218502_s_at | 7227 | *TRPS1* | -2.33 | 1.51E-03 | -2.34 | 2.42E-03 |
|  | 222651_s_at | 7227 | *TRPS1* | -1.63 | 1.17E-02 | -2.13 | 4.53E-03 |
|  | 224048_at | 84101 | *USP44* | -1.30 | 3.17E-02 | -1.41 | 3.68E-02 |
|  | 214218_s_at | 7503 | *XIST* | -1.69 | 5.42E-04 | -2.24 | 4.37E-02 |
|  | 224588_at | 7503 | *XIST* | -1.80 | 2.96E-02 | -2.77 | 4.39E-02 |
|  | 215540_at | 10730 | *YME1L1* | -1.60 | 1.17E-02 | -1.68 | 1.29E-02 |
|  | 215796_at | 10730 | *YME1L1* | -1.89 | 9.20E-03 | -3.46 | 2.13E-04 |
|  | 216133_at | 10730 | *YME1L1* | -2.91 | 3.65E-04 | -3.54 | 1.70E-04 |
|  | 217143_s_at | 10730 | *YME1L1* | -2.49 | 9.70E-04 | -3.97 | 8.08E-05 |
|  | 217397_at | 10730 | *YME1L1* | -1.37 | 2.49E-02 | -1.76 | 9.78E-03 |
|  | 234013_at | 10730 | *YME1L1* | -3.18 | 1.91E-04 | -3.74 | 1.45E-04 |
|  | 234402_at | 10730 | *YME1L1* | -1.76 | 7.77E-03 | -1.31 | 3.79E-02 |
|  | 234427_at | 10730 | *YME1L1* | -1.58 | 1.30E-02 | -1.93 | 8.85E-03 |
|  | 234849_at | 10730 | *YME1L1* | -2.39 | 2.27E-03 | -3.39 | 2.20E-04 |
|  | 234964_at | 10730 | *YME1L1* | -2.65 | 6.20E-04 | -3.11 | 5.29E-04 |
|  | 230224_at | 644353 | *ZCCHC18* | -2.05 | 3.17E-03 | -1.35 | 2.99E-02 |
|  | 1562529_s_at | NA | *NA* | -1.27 | 3.50E-02 | -1.61 | 1.79E-02 |
|  | 213416_at | NA | *NA* | -2.98 | 3.20E-04 | -2.73 | 2.57E-03 |
|  | 214945_at | NA | *NA* | -1.98 | 3.70E-03 | -1.37 | 2.49E-02 |
|  | 215262_at | NA | *NA* | -1.94 | 4.39E-03 | -1.26 | 4.94E-02 |
|  | 215388_s_at | NA | *NA* | -2.41 | 1.20E-03 | -2.71 | 1.32E-03 |
|  | 215524_x_at | NA | *NA* | -2.23 | 1.86E-03 | -2.69 | 1.08E-03 |
|  | 216191_s_at | NA | *NA* | -2.53 | 8.37E-04 | -4.19 | 3.78E-05 |
|  | 217394_at | NA | *NA* | -3.69 | 3.81E-05 | -1.36 | 8.89E-03 |
|  | 217412_at | NA | *NA* | -1.33 | 1.37E-02 | -3.92 | 8.17E-05 |
|  | 224533_s_at | NA | *NA* | -1.58 | 2.83E-02 | -4.36 | 3.03E-05 |
|  | 227556_at | NA | *NA* | -1.25 | 4.47E-02 | -1.35 | 3.79E-02 |
|  | 234396_at | NA | *NA* | -2.67 | 6.10E-04 | -3.20 | 3.40E-04 |
|  | 234842_at | NA | *NA* | -1.98 | 3.65E-03 | -1.70 | 1.31E-02 |
|  | 234848_at | NA | *NA* | -1.28 | 3.45E-02 | -1.91 | 9.03E-03 |
|  | 234860_at | NA | *NA* | -1.70 | 8.73E-03 | -2.15 | 5.79E-03 |
|  | 234867_at | NA | *NA* | -1.94 | 4.16E-03 | -2.12 | 4.44E-03 |
|  | 234883_x_at | NA | *NA* | -2.16 | 2.22E-03 | -1.90 | 7.57E-03 |
|  | 234886_at | NA | *NA* | -1.71 | 9.27E-03 | -2.45 | 2.12E-03 |
|  | 236704_at | NA | *NA* | -1.23 | 4.19E-02 | -2.18 | 4.24E-03 |
|  | 243366_s_at | NA | *NA* | -2.65 | 6.36E-04 | -2.52 | 5.35E-03 |
|  | 243602_at | NA | *NA* | -2.80 | 4.68E-04 | -1.59 | 9.73E-03 |
|  | 243810_at | NA | *NA* | -1.61 | 1.21E-02 | -2.24 | 5.09E-03 |
